# Mesenchymal stem cells exosomal let-7a-5p improve autophagic flux and alleviate liver injury in acute-on-chronic liver failure by promoting nuclear expression of TFEB

**DOI:** 10.1038/s41419-022-05303-9

**Published:** 2022-10-12

**Authors:** Dengna Lin, Hao Chen, Jing Xiong, Jing Zhang, Zhaoxia Hu, Juan Gao, Bin Gao, Shaoquan Zhang, Junfeng Chen, Huijuan Cao, Zhihui Li, Bingliang Lin, Zhiliang Gao

**Affiliations:** 1 Department of Infectious Diseases, the Third Affiliated Hospital of Sun Yat-Sen University, Guangzhou, China; 2 Guangdong Provincial Key Laboratory of Liver Disease Research, the Third Affiliated Hospital of Sun Yat-Sen University, Guangzhou, China; 3 Department of Neurology, the First Affiliated Hospital of Nanchang University, Nanchang, China; 4 Key Laboratory of Tropical Disease Control, Sun Yat-Sen University, Ministry of Education, Guangzhou, China

**Keywords:** Autophagy, Mesenchymal stem cells

## Abstract

Acute-on-chronic liver failure is a distinct clinical syndrome characterized by a dysregulated immune response and extensive hepatocyte death without satisfactory therapies. As a cytoplasmic degradative and quality-control process, autophagy was implicated in maintaining intracellular homeostasis, and decreased hepatic autophagy was found in many liver diseases and contributes to disease pathogenesis. Previously, we identified the therapeutic potential of mesenchymal stem cells (MSCs) in ACLF patients; however, the intrinsic mechanisms are incompletely understood. Herein, we showed that MSCs restored the impaired autophagic flux and alleviated liver injuries in ACLF mice, but these effects were abolished when autophago-lysosomal maturation was inhibited by leupeptin (leu), suggesting that MSCs exerted their hepatoprotective function in a pro-autophagic dependent manner. Moreover, we described a connection between transcription factor EB (TFEB) and autophagic activity in this context, as evidenced by increased nuclei translocation of TFEB elicited by MSCs were capable of promoting liver autophagy. Mechanistically, we confirmed that let-7a-5p enriched in MSCs derived exosomes (MSC-Exo) could activate autophagy by targeting MAP4K3 to reduce TFEB phosphorylation, and MAP4K3 knockdown partially attenuates the effect of anti-let-7a-5p oligonucleotide via decreasing the inflammatory response, in addition, inducing autophagy. Altogether, these findings revealed that the hepatoprotective effect of MSCs may partially profit from its exosomal let-7a-5p mediating autophagy repairment, which may provide new insights for the therapeutic target of ACLF treatment.

## Introduction

Acute-on-chronic liver failure (ACLF) is featured with acute decompensation of liver function in patients with chronic liver diseases. Unlike decompensated cirrhosis, ACLF is characterized by submassive or massive hepatocyte necrosis/apoptosis and uncontrolled systemic inflammation, followed by multiple organ failures and extremely high mortality within 28 days [1, 2]. Despite extensive efforts and significant improvement in ACLF treatment, the conventional therapeutic interventions, including nucleoside antiviral drugs, artificial liver support system, and liver transplantation, are not yet satisfactory due to the poor response and lack of donor livers [3–5]. Thus, searching for more effective treatment strategies has become an urgent issue to be resolved.

In recent years, significant attention has focused on the potential use of mesenchymal stem cells (MSCs) for improving the treatment of inflammatory and degenerative diseases due to their unique immune-regulatory and regenerative capacity [6, 7]. Our previous study showed that MSCs transplantation could obviously improve the short-term survival rate and reduce the incidence of complications such as severe infection in ACLF patients [8], but certain obstacles (e.g., low cell survival and migrating rate, cell senescence) remain to be overcome [9–12]. As newly discovered 30–120 nm small diameter vesicles, exosomes are thought to be secreted by MSCs and mediate their therapeutic effect through transferring contents of miRNA and proteins to the recipient cells. In addition, compared with MSCs, exosomes have the same immune-modulative function and gain the advantage of liver distribution, avoiding the problem of aging or rejection in the application of its cellular counterparts [13–16]. Therefore, the determination of hepatoprotective components in exosomes derived from MSCs (MSC-Exo) may provide a new strategy for ACLF treatment.

Autophagy, is an evolutionarily conservative cellular adaptive response against intra- or extracellular stress or stimuli, by which damaged organelles or misfolded proteins were degraded and recycled for ATP production and protein synthesis to facilitate cell survival and homeostasis [17]. Previous studies indicated that under conditions of mild hepatic injury, the autophagic signaling cascade can be activated to protect cells from death; however, in the case of more severe or prolonged liver damage, autophagy seems to be inhibited [18], which is contradictory to the view that autophagy would be quickly induced under pathological conditions. In this regard, if and how the autophagic function is affected under the condition of ACLF is not clear and needs to be further explored. Meanwhile, multiple studies demonstrated the hepatoprotective effect of MSCs transplantation could be mediated by regulating autophagy and reducing the inflammatory response in case of liver fibrosis or acute liver injury [19, 20]; however, whether autophagy is involved in the protective effect of MSCs therapy for ACLF and the specific mechanism is not known.

Hence, in this study, we aim to investigate whether the biological effect of MSCs therapy on ACLF was due to the regulation of autophagy and determine the related molecular mechanism. Finally, we present evidence that the administration of MSCs restored the impaired autophagic flux by inducing the formation of autolysosomes and therefore protecting hepatocytes from death in vivo in ACLF mice. Mechanistically, we identified that the let-7a-5p, which is enriched in exosomes, could mediate the autophagy regulation of MSCs by promoting the nuclear translocation of transcription factor EB (TFEB) and inducing the expression of lysosome genes.

## Materials and methods

### Animal management and establishment of the ACLF model

All animal experiments applied in this study were conducted with the approval of the Laboratory Animal Ethics Committee of Guangzhou Forevergen Biosciences. C57BL/6, B6 mice (male, 4–6 weeks, weighting 18–22 g) were purchased from the Guangdong Medical Laboratory Animal Center (Guangdong, China) and assigned randomly to groups. All mice were housed in specific pathogen-free conditions and exposed to a 12 h daylight/darkness environment, allowing unlimited access to food and water. To establish of ACLF model, the mice were intraperitoneally injected with 10% carbon tetrachloride (CCl4, #319961, Sigma, USA, 5 ml/kg body weight) twice a week for 8 consecutive weeks, and challenged with a single dose of 50% carbon tetrachloride (8 ml/kg) in day 3 of week 8 (ACLF group). The control mice were intraperitoneally injected with the same amount of olive oil at the same time (Sham group). After the establishment of the ACLF model, mice were further divided into two groups, which were infused with MSCs (1 × 10^5^, resuspended in sterile saline (NS)) (ACLF + MSC group) or saline via tail vein. For in vivo autophagy flux measurements, we intraperitoneally injected mice with Leu (#HY-18234A, MCE, USA, 20 mg/kg body weight) or phosphate buffer solution (PBS) [21].

### Preparation and characterization of MSCs and exosomes

The MSCs (human bone marrow mesenchymal stem cells) were obtained from the Human Center for Stem Cell Biology and Tissue Engineering, Key Laboratory for Stem Cells and Tissue Engineering, Guangzhou, Guangdong, China. The identification and characterization methods of MSCs were performed as previously described [6]. Exosomes were isolated by ultracentrifugation and analyzed for nanoparticle tracking analysis (NTA), and the protein markers and morphology were detected by western blot and scanning electron microscope (SEM), respectively (Fig. S1A and S1C). In brief, MSCs in the fifth generation were seeded in T75 cm^2^ flasks and cultured in low-glucose Dulbecco’s modified Eagle medium (DMEM) with 10% fetal bovine serum (FBS) and 100 ug/mL penicillin-streptomycin. After reaching 50–60% confluence, MSCs were washed by PBS, then the fresh low-glucose DMEM with exosome-depleted FBS was added and cultured for another 48 h. The collected supernatant was centrifuged at 2000 × *g* for 20 min at 4 °C and then 10,000 × *g* for 40 min at 4 °C to remove cell debris and separate microvesicles. Then, the supernatant was transferred to ultracentrifuge tubes and centrifuged twice at 100,000 × *g* for 90 min at 4 °C to obtain exosomes [22]. Exosomes were resuspended in 100 µL PBS and stored at −80 °C.

### Cell culture and establishment of CCl4-injury (C/I) model

A normal human liver cell line of *L02* cells was purchased from iCell Bioscience Inc (Shanghai, China) and cultured in high-glucose DMEM with 10% FBS and 100ug/mL penicillin-streptomycin. To the establishment of in vitro *C/I* model, which mimics the severity of liver injury in ACLF, *L02* cells were treated with 50 μM carbon tetrachloride for 3 h (C/I group), during performing autophagic flux assay, 100 μM leupeptin was added to block lysosomal proteolysis at 4 h before protein extraction [23]. For the in vitro MSCs and exosome treatment experiment, MSCs were inoculated in the upper transwell chambers (0.4 μm, Corning, USA) of six-well plates and co-cultured with *L02* cells at a 2:1 ratio for 18 h (C/I + MSC group) (Fig. S1D). Exosomes were added directly to the culture medium of *L02* cells at a ratio of 2 ug /1 × 10^5^ recipient cells and cultured for another 18 h (C/I + MSC-Exo group). To prevent exosome secretion, MSCs were pretreated with the phospholipase inhibitors GW4869 (20 μM) (#D1692, Sigma, USA) for 12 h.

### Statistical analysis

All data were expressed as Mean ± SD, the student *t*-test was applied to compare two groups of quantitative data, while three or more groups of quantitative data were compared by One-way ANOVA combined with Bonferroni multiple comparison test. *P*-values < 0.05 were considered statistically significant, and SPSS 22.0 and GraphPad Prism 8.0 were used for statistical analysis.

## Results

### MSCs administration alleviates liver injury and repairs autophagic flux in ACLF mice

**Fig. 1:**
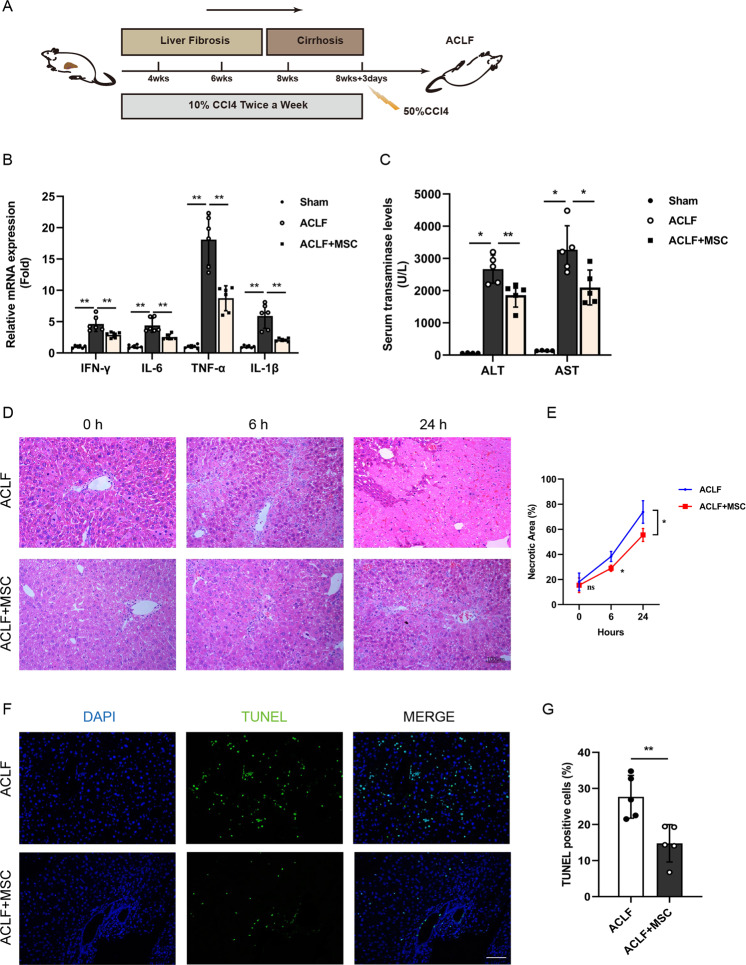
MSCs administration alleviates liver injury in ACLF mice. **A** Schematic timeline for the generation of ACLF mice by injected intraperitoneally with carbon tetrachloride, MSCs (10^5^ per mouse, resuspended in sterile saline) were infused at the time of acute CCl4 administration. **B** mRNA levels of IFN-γ, IL-6, TNF-α, IL-1β (normalized to GAPDH) in hepatocytes from normal group (Sham group) and ACLF mice treated with either vehicle buffer or MSCs were measured at 6 h after transfusion by qRT-PCR (*n* = 6 per group). Data represent mean ± SD, **p < 0.01, one-way ANOVA with Bonferroni’s post analyses. **C** The Serum transaminases ALT and AST levels were quantified in sham group and ACLF mice treatment with or without MSCs for 6 h (*n* = 5 except for sham group, *n* = 4). Data represent mean ± SD, *p < 0.05, **p < 0.01, one-way ANOVA with Bonferroni’s post analyses. **D** H&E staining of liver tissue from ACLF mice 0, 6, and 24 h after vehicle buffer or MSCs treatment. ×200 magnification, scale bar, 100 µm. Representative images were presented from *n* = 4 mice per group. **E** Quantification of the percentage of necrotic area from indicated two groups. Data represent mean ± SD, ns nonsignificant, *p < 0.05, Student’s *t*-test was performed at individual time periods. **F** Representative images of TUNEL IF staining showed cell death in liver tissue of ACLF mice treatment with or without MSCs for 6 h. Green, TUNEL-positive; blue, DAPI; ×200 magnification, scale bar, 100 µm. **G** Quantitative analysis of the percentage of TUNEL-positive cells from indicated two groups (*n* = 5 per group). Data represent mean ± SD, **p < 0.01, Student’s *t*-test was performed.

We previously identified that MSCs can improve the survival rate of ACLF patients. In order to investigate whether the improvement of ACLF by MSCs transplantation was associated with autophagic activity modulation, we first established a murine ACLF model which mimics the pathogenesis of clinical settings by administering CCl4 (Fig. 1A) and evaluated the therapeutic effects of MSCs. In macroscopic views, the liver tissue of mice in the model group exhibited a brown appearance, uneven surface with nodular protrusions of different sizes, and a hard texture (Fig. S1E). Histopathological analysis of the liver of ACLF mice revealed disrupted architecture, obvious fibrosis, massive hepatocyte necrosis, and portal/periportal inflammation occurred throughout the lobules, as we previously reported [24]. As shown in Figs. S1F and 1G, MSCs were successfully isolated, osteogenic and lipogenic differentiation was induced, and flow cytometry verified that MSCs used in our study were concordant with the definition of MSCs [6]. Next, we evaluated the hepatoprotective effect of MSCs, and found that significantly reduced mortality of ACLF mice, visually decreased mRNA expression of TNF-α, IFN-γ, IL-6, and IL-1β in the liver (Fig. 1B), and serum AST and ALT levels can be seen after 24 h of MSCs administration (Fig. 1C). Furthermore, the hepatic necrosis in the lobules was also significantly mitigated by MSCs transplantation, which can be seen at 6 h and became more evident at 24 h (Fig. 1D, E), in lines with H&E staining, the result of TUNEL assay confirmed that cell death rate was markedly reduced after 6 h of MSCs therapy. (Fig. 1F, G).

**Fig. 2:**
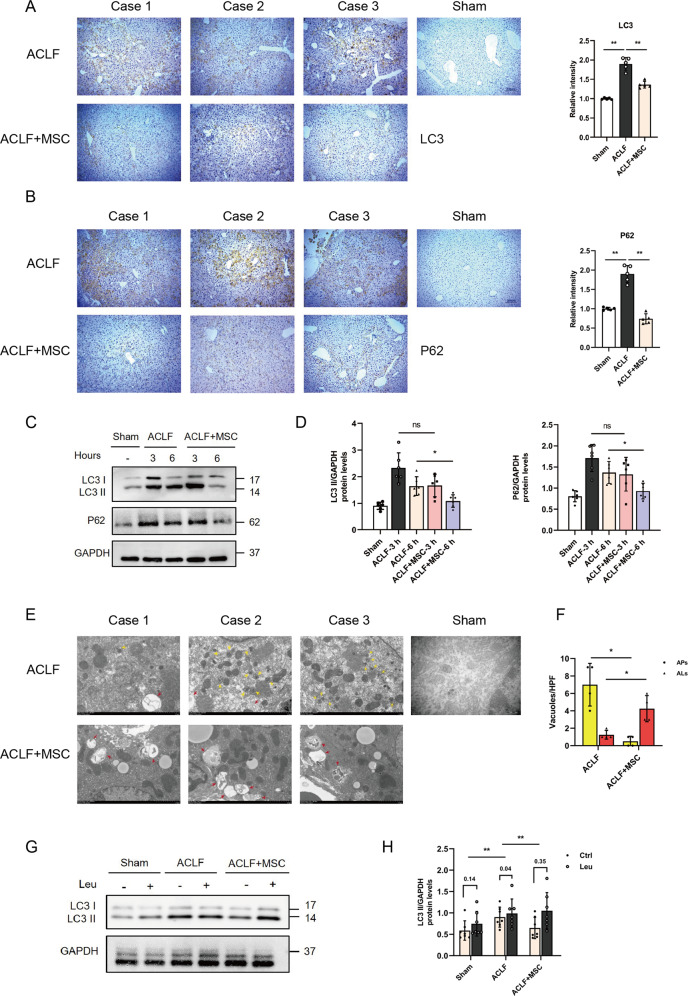
MSCs administration repair autophagic flux in ACLF mice. **A**, **B** Immunohistochemical staining for LC3 and P62 in liver sections from ACLF mice treatment with either vehicle buffer or MSCs for 6 h. Representative images were presented from *n* = 5 mice per group and statistic of IHC staining for LC3 and P62 in liver tissues were quantified in the adjacent panel. ×100 magnification, scale bar, 200 µm. Data represent mean ± SD, **p < 0.01, one-way ANOVA with Bonferroni’s post analyses. **C**, **D** Protein levels of LC3 and P62 (normalized to GAPDH) in hepatocytes from ACLF mice treated with either vehicle buffer or MSCs were measured at 3 h and 6 h after transfusion by western blot. Representative images were presented and bar graphs are quantified results of relative gray value in protein bands by Image J (*n* = 6 per group). Data represent mean ± SD, ns nonsignificant, *p < 0.05, one-way ANOVA with Bonferroni’s post analyses. **E**, **F** Transmission electron micrographs of liver sections from ACLF mice treatment with either vehicle buffer or MSCs for 6 h, representative images were presented from *n* = 4 mice per group and the number of autophagosomes (APs) and autolysosomes (ALs) vacuoles was calculated. ×6000 magnification, scale bar, 2 µm. Yellow arrows: autophagosomes (APs), red arrows: autolysosomes (ALs). *p < 0.05, Student’s *t*-tests were performed. **G**, **H** Leupeptin (Leu) was used to evaluate the autophagic flux, LC3 II protein levels both before and after Leu intervention were measured in hepatocytes of sham group and ACLF mice treated with either vehicle buffer or MSCs at 6 h after transfusion by western blot. Leu (20 mg/kg) was intraperitoneal injected 4 h before sacrifice. Both of LC3 II protein levels before and after Leu intervention were quantified, and the absolute changes (indicating autophagic flux) were calculated and analyzed by one-way ANOVA with Bonferroni’s post analyses (*n* = 6–7 per group). Data represent mean ± SD, *p < 0.05.

To examine the effect of MSCs on autophagy, we first analyzed the molecular changes in the autophagy proteins LC3 (a marker of autophagosomes) and p62 (autophagic degrade substrates). Notably, the IHC staining in liver tissue of ACLF mice showed that the levels of LC3 and p62 were obviously decreased after 6 h of MSCs transfusion (Fig. 2A, B). Moreover, the results of the western blot also revealed a significant reduction in LC3 and p62 expression has occurred after 6 h of MSCs treatment (Fig. 2C, D). The lack of p62 accompanied by reduced expression of LC3-II indicated enhanced autophagic degradation activity in the late stages of autolysosome processing [25].

### MSCs facilitates the fusion of autophagosomes with lysosomes in the acute injury of ACLF

To verify the late stage of autophagy, which autophagosomes were fused with lysosomes to form autolysosomes, was promoted by MSCs, and transmission electron micrography (TEM) was performed [25]. As shown in Fig. 2E, F, compared to ACLF group, the number of autolysosomes in the liver was substantially increased, and autophagosomes decreased in ACLF + MSC group. In addition, the pharmacological flux assays using protease inhibitor leupeptin to block the formation of autolysosomes also showed the LC3-II accumulation (Fig. 2G, H) was significantly greater in ACLF + MSC group than ACLF group. These results are indicative of autolysosome formation being blocked in ACLF and thus leading to the accumulation of autophagosomes, and the above scenarios were gradually reversed after administration of MSCs [25].

**Fig. 3:**
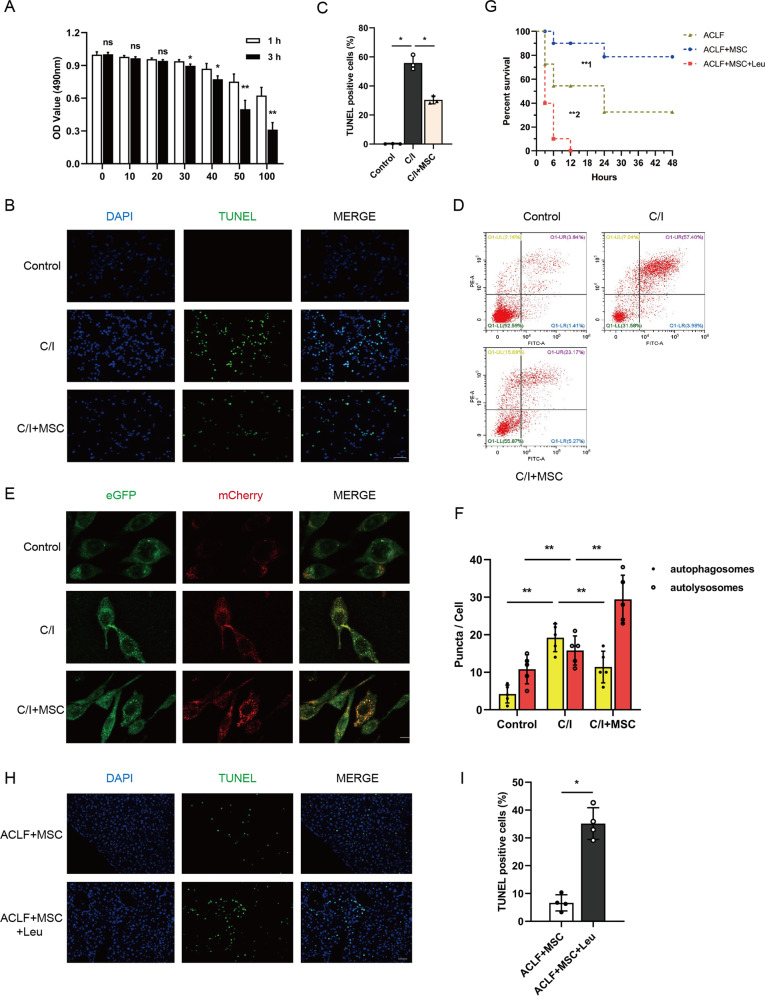
Blockade of autophagy-lysosome flux impaired the hepatoprotective effect of MSCs. **A** L02 cells treated with different concentration (0–100 µM) of carbon tetrachloride for 0 and 3 h, and then cell viability was detected by cell counting kit-8 (CCK8) assay and time-dependent viability reduction was analyzed by student’s *t*-test at each concentration point (*n* = 5 per group). Data represent mean ± SD, ns nonsignificant, *p < 0.05, **p < 0.01. **B**, **C** Representative images of TUNEL IF staining showed cell death in control group and 3 h C/I exposed L02 cells with or without MSCs co-culture and quantification of the percentage of TUNEL-positive cells from indicated three groups by one-way ANOVA with Bonferroni’s post analyses (*n* = 5 per group). Green, TUNEL-positive; blue, DAPI; ×200 magnification, scale bar, 100 µm. Data represent mean ± SD, *p < 0.05. **D** L02 cells in control group and 3 h C/I exposed L02 cells with or without MSCs co-culture groups were stained with Annexin-V/PI and percentage (%) cell death (Annexin-V^**+**^/PI^**−**^ and Annexin V^**+**^/PI^**+**^ cells) is shown. **E**, **F** Representative immunofluorescence images of mCherry-eGFP-LC3 transfected L02 cells in groups of control and 3 h C/I exposed L02 cells with or without MSCs co-culture were visualized by confocal microscopy and the number of eGFP^**+**^/mCherry^**+**^ (yellow, indicated autophagosomes) and eGFP^**-**^/mCherry^**+**^ (red) was calculated (*n* = 5 per group). ×630 magnification, scale bar, 20 µm. Data represent mean ± SD, **p < 0.01, one-way ANOVA with Bonferroni’s post analyses. **G** Kaplan–Meier survival curves for ACLF mice bearing vehicle buffer or MSCs treatment challenged with or without Leu (*n* = 10 per group). Data represent mean ± SD, **p < 0.01 (**1 indicated comparison between groups of ACLF mice bearing vehicle buffer or MSCs treatment, **2 indicated comparison between groups of ACLF mice receiving MSCs treatment challenged with or without Leu), Gehan–Breslow–Wilcoxon tests were performed. **H**, **I** Representative images of TUNEL IF staining showed cell death in ACLF mice bearing MSCs treatment challenged with or without Leu and quantification of the percentage of TUNEL-positive cells from indicated two groups by Student’s *t*-test (*n* = 4 per group). Green, TUNEL-positive; blue, DAPI; ×200 magnification, scale bar, 100 µm. Data represent mean ± SD, *p < 0.05.

Multiple studies demonstrated the application of mCherry-eGFP-LC3 construct could monitor the autophagic flux by detecting the relative intensity of red/green light spots, in brief, the yellow puncta were a combination of GFP and RFP fluorescence which represent autophagosomes, whereas red puncta whose acidic pH quenches GFP fluorescence represent autolysosomes [26, 27]. For a further intuitively observation of the effect of MSCs on autophagic flux, an in vitro model that mimics the severity of liver injury in ACLF was established (C/I model), Based on the result of half lethal concentration (LD50), we chose a 3 h treatment with carbon tetrachloride 50 µM as the optimal condition for subsequent experiments (Fig. 3A). In accordance with in vivo experiments, the result of TUNEL assay and flow cytometry demonstrated that cell death was reduced in MSCs co-cultured C/I L02 cells (Figs. 3B through 3D), furthermore, mRNA levels of TNF-α, IFN-γ, IL-6 and IL-1β in cells were also markedly decreased in *C/I* + MSC group (Fig. S1H). Notably, the results of genetic flux assays using mCherry-eGFP-LC3 lentivirus transfected *L02* hepatocytes showed persistence of green puncta in the condition of lethal liver injury, administration of MSCs significantly increased the red puncta and resulted in the quenching of the green fluorescence (Fig. 3E, F), suggested the late stage of autophagy was repaired, and fusion of autophagosomes into lysosomes was promoted by MSCs.

### Inhibiting the formation of autolysosomes impaired the hepatoprotective effect of MSCs in ACLF mice

To determine the hepatoprotective effects of MSCs were dependent on autophagic flux regulation, we next treated each group of mice with leupeptin (Leu), a protease inhibitor that blocked the formation of autolysosomes, and detected the change in liver injury and hepatocellular death. Apart from the sharply reduced survival rates (Fig. 3G), administration of Leu could significantly reverse the effect of MSCs on serum levels of transaminase (AST and ALT) and mRNA levels of TNF-α, IFN-γ, IL-6, and IL-1β in the liver (Fig. S1I and S1J). Moreover, results of TUNEL assay showed the number of dead cells was obviously increased after Leu treatment (Fig. 3H, I), suggesting the therapeutic effect of MSCs in ACLF mice was dampened by inhibiting the formation of autolysosomes.

### MSCs repair autophagic flux by promoting TFEB nuclear translocation

**Fig. 4:**
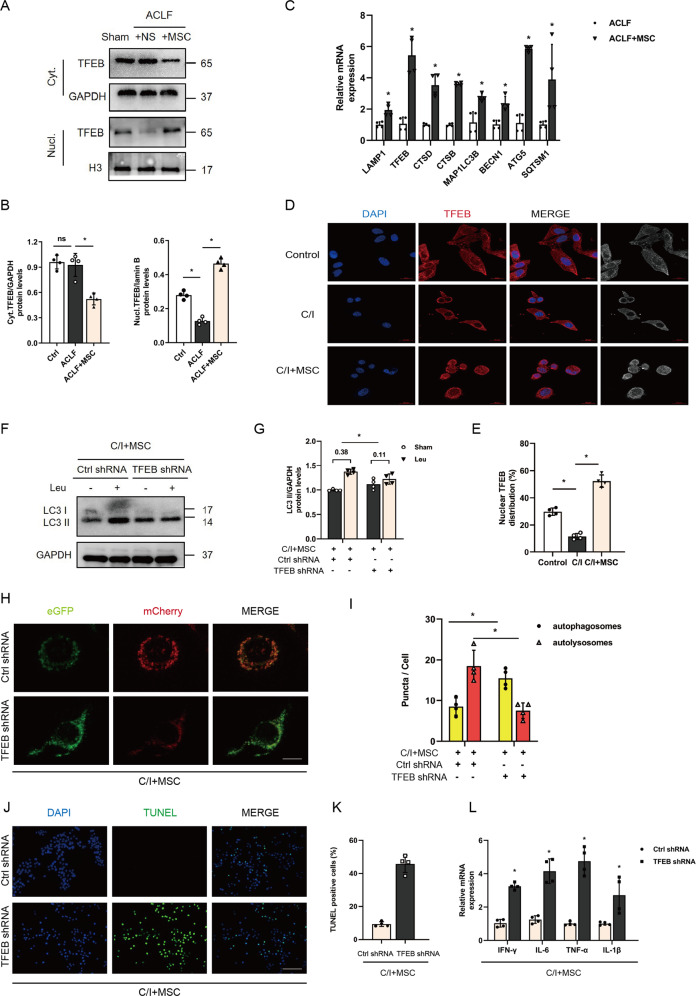
MSCs regulate autophagic flux via inducing TFEB nuclei translocation. **A**, **B** Nuclear and cytoplasmic TFEB protein levels in hepatocytes of sham group mice or ACLF mice receiving either vehicle buffer or MSCs transplantation for 6 h were measured by western blot. GAPDH and Histone H3 were used as loading controls for cytoplasmic and nuclear fractions, respectively. Representative images were presented and bar graphs are quantified results of relative gray value in protein bands by Image J (*n* = 4 per group). Data represent mean ± SD, ns nonsignificant, *p < 0.05, one-way ANOVA with Bonferroni’s post analyses. **C** mRNA levels of LAMP1, TFEB, CTSD, CTSB, MAP1LC3B, BECN1, ATG5, SQSTM1(normalized to GAPDH) in hepatocytes of ACLF mice treated with either vehicle buffer or MSCs were measured at 6 h after transfusion by qRT-PCR (*n* = 4 per group). Data represent mean ± SD, *p < 0.05, student’s *t*-test was performed. **D**, **E** Immunofluorescent staining of TFEB in control group L02 cells and 3 h C/I exposed L02 cells with or without MSCs co-culture showed the subcellular localization of TFEB, representative images were presented (*n* = 4 per group) and red channel fluorescence images which indicated TFEB were converted to white and black and listed in the rightmost column for clearer contrasts of different subcellular compartments. Measurement of nuclear TFEB fluorescence intensity was calculated by percentage of total fluorescence. Red, TFEB; blue, DAPI; ×630 magnification, scale bar, 20 µm. Data represent mean ± SD, *p < 0.05, one-way ANOVA with Bonferroni’s post analyses. **F**, **G** L02 cells were infected with TFEB shRNA or Ctrl shRNA, and then bearing 3 h C/I with MSCs co-culture. 100 μM leupeptin was added to block lysosomal proteolysis for autophagic flux assay at 4 h before protein extraction. LC3 II protein levels in L02 cells of the above indicated groups both before and after Leu intervention were measured by western blot. Both of LC3 II protein levels before and after Leu intervention were quantified, and the absolute changes indicating autophagic flux (*n* = 4 per group). Data represent mean ± SD, *p < 0.05. Student’s *t*-test was performed. **H**, **I** Representative immunofluorescence images of mCherry-eGFP-LC3 transfected L02 cells from above two groups indicated in **F**, **G** were visualized by confocal microscopy and the number of eGFP + /mCherry + (yellow, indicated autophagosomes) and eGFP-/mCherry + (red) was calculated (*n* = 4 per group). ×630 magnification, scale bar, 20 µm. Data represent mean ± SD, *p < 0.05, Student’s *t*-test was performed. **J**, **K** Representative images of TUNEL IF staining showed cell death in L02 cells from above two groups and percentage of TUNEL-positive cells were quantified by Student’s *t*-test (*n* = 4 per group). Green, TUNEL-positive; blue, DAPI; ×200 magnification, scale bar, 100 µm. Data represent mean ± SD, *p < 0.05. **L** mRNA levels of IFN-γ, IL-6, TNF-α, IL-1β (normalized to GAPDH) in L02 cells from the above two groups were measured by qRT-PCR (*n* = 4 per group). Data represent mean ± SD, *p < 0.05, Student’s *t*-test was performed.

To understand the mechanism by which MSCs enhanced the synthesis and function of autolysosomes, we then performed western blot to detect the nuclear and cytoplasmic expression of transcriptional factor EB (TFEB), which was shown to regulate lysosomal biogenesis and autophagy upon its translocation into the nucleus and activate the transcription of the related genes [25, 28]. In our study, we found that transfusion of MSCs could increase nuclear translocation of TFEB in ACLF primary hepatocytes (Fig. 4A, B). Notably, as shown in Fig. 4C, MSCs administration could induce the expression of lysosomes and autophagy-related genes, which seems to be partially contradicted by the expression of LC3-II protein reported above but laterally reflected the enhancement of degradative function and involvement of TFEB. Additionally, in vitro data of fluorescence staining validated that MSCs co-culture can stimulate the nuclear translocation of TFEB in C/I cells (Fig. 4D, E).

To specifically verify whether TFEB is required for the MSC-mediated repairment of autophagic flux, we silenced TFEB in *L02* hepatocytes by transfecting with TFEB shRNA adenovirus. As shown in Fig. 4F through I, TFEB silencing not only attenuated MSCs-induced accumulation of LC3-II proteins with leupeptin but also reduced the number of red puncta in mCherry-eGFP-LC3 transfected *L02* hepatocytes co-cultured with MSCs. Furthermore, the number of TUNEL-positive cells and mRNA levels of TNF-α, IFN-γ, IL-6, and IL-1β in C/I + MSCs group were obviously increased after TFEB was knockdown (Fig. 4J through L). These suggested that TFEB is a critical mediator for MSCs repairs the autophagic flux, and alleviates liver injury in ACLF.

### Let-7a-5p is enriched in MSC-secreted exosomes and induces TFEB nuclear translocation

**Fig. 5:**
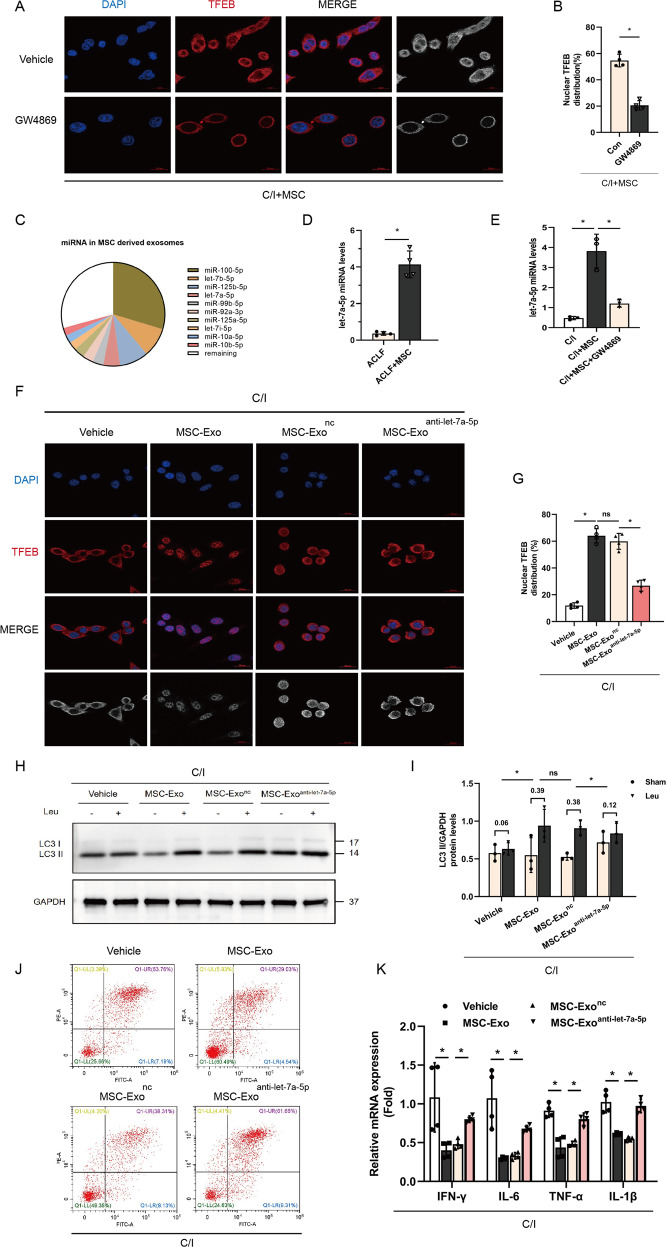
let-7a-5p in MSCs derived exosomes (MSC-Exo) restore autophagic flux via inducing TFEB nuclei translocation. **A**, **B** MSCs were pretreated with serum-free DMEM containing phospholipase inhibitors GW4869 (20 μM) or vehicle buffer (DMSO 0.1%) for 12 h, and then co-cultured with L02 cells bearing 3-h C/I exposure. Immunofluorescent staining of TFEB in the above indicated two groups showed the subcellular localization of TFEB, representative images were presented (*n* = 4 per group) and red channel fluorescence images which indicated TFEB were converted to white and black and listed in the rightmost column for clearer contrasts of different subcellular compartments. Measurement of nuclear TFEB fluorescence intensity was calculated by percentage of total fluorescence. Red, TFEB; blue, DAPI; ×630 magnification, scale bar, 20 µm. Data represent mean ± SD, *p < 0.05, one-way ANOVA with Bonferroni’s post analyses. **C** Pie chart of active individual miRNA in MSC-Exo, percentage of reads mapping to miRNA are shown for the ten most abundant miRNA. **D** miRNA levels of let-7a-5p (normalized to U6) in hepatocytes of ACLF mice treated with either vehicle buffer or MSCs were measured at 6 h after transfusion by qRT-PCR (*n* = 4 per group). Data represent mean ± SD, *p < 0.05, student’s *t*-test was performed. **E** MSCs were pretreated with GW4869 or vehicle buffer as above, and then co-cultured with L02 cells bearing 3 h C/I exposure, miRNA levels of let-7a-5p (normalized to U6) in L02 cells without MSCs treatment and the above two co-cultured groups were measured by qRT-PCR (*n* = 4 per group). Data represent mean ± SD, *p < 0.05, one-way ANOVA with Bonferroni’s post analyses. **F**, **G** Obtained MSC-Exo were pretreated with let-7a-5p inhibitor (MSC-Exo^anti-let-7a-5p^) or a negative control oligonucleotide (MSC-Exo^nc^), and then the obtained MSC-Exo, MSC-Exo^nc^, and MSC-Exo^anti-let-7a-5p^ were co-cultured with L02 cells bearing 3 h C/I exposure, immunofluorescence staining for TFEB in L02 cells bearing 3 h C/I exposure alone and the above three co-cultured groups was performed to show the subcellular localization of TFEB, representative images were presented (*n* = 4 per group) and red channel fluorescence images which indicated TFEB were converted to white and black and listed in the rightmost column for clearer contrasts of different subcellular compartments. Measurement of nuclear TFEB fluorescence intensity was calculated by percentage of total fluorescence. Red, TFEB; blue, DAPI; ×630 magnification, scale bar, 20 µm. Data represent mean ± SD, *p < 0.05, one-way ANOVA with Bonferroni’s post analyses. **H**, **I** After L02 cells in the above four groups were incubated with or without leupeptin (Leu) for 4 h, proteins were extracted and LC3 II protein levels both before and after Leu intervention were measured by western blot. Both of LC3 II protein levels before and after Leu intervention were quantified, and the absolute changes indicating autophagic flux (*n* = 3 per group). Data represent mean ± SD, ns nonsignificant, *p < 0.05. One-way ANOVA with Bonferroni’s post analyses. **J** L02 cells in the above four groups were stained with Annexin-V/PI and percentage (%) cell death (Annexin-V^**+**^/PI^**−**^ and Annexin V^**+**^/PI^**+**^ cells) is shown. **K** mRNA levels of IFN-γ, IL-6, TNF-α, IL-1β (normalized to GAPDH) in L02 cells from above four groups were determined by qRT-PCR. (*n* = 4 per group). Data represent mean ± SD, *p < 0.05, One-way ANOVA with Bonferroni’s post analyses.

Next, we investigated whether the increase in TFEB nuclei expression after transfusion of MSCs is partially mediated by mesenchymal stem cell (MSC) derived exosomes (MSC-Exo), GW4869 sphingomyelinase inhibitors were applied to block the secretion of exosomes from MSCs and found an obviously decreased nuclear translocation of TFEB in co-cultured *L02* hepatocytes administered GW4869 (Fig. 5A, B). To determine the components of exosomes responsible for MSC-Exo regulating TFEB expression, exosomal miRNA-seq analyses were performed to select the candidate miRNA. The seq-data suggested that miR-100-5p, let-7a/b/i-5p, miR-125a/b-5p, miR-99b-5p, miR-92a-5p, and miR-10a/b-5p were the top ten enriched miRNAs in MSC-Exo (Table S2 and Fig. 5C). Among of the miRNAs mentioned above, let-7a-5p was lately reported to significantly decreased in ACLF patients and associated with 30-day mortality of patients [29]. We, therefore, supposed that the transfer of let-7a-5p to the recipient hepatocytes by exosomes may mediate the therapeutic effect of MSCs, and thus the regulatory role of let-7a-5p on TFEB nuclear translocation and autophagic flux was addressed.

As shown in Fig. 5D, the results of quantitative polymerase chain reaction analyses validated that let-7a-5p was reduced in ACLF mice, and MSCs treatment can significantly upregulate its expression in primary hepatocytes of ACLF mice. Furthermore, in vitro co-culture model revealed that the levels of let-7a-5p in *L02* cells with MSCs co-cultured markedly decreased after applying GW4869 (Fig. 5E), which indicated that MSCs increased levels of let-7a-5p in ACLF was partially through the transfer of exosomes.

To determine if there is an association between the increased let-7a-5p levels and nuclear translocation of TFEB and autophagic flux repairment, we first pretreated MSC-Exo with let-7a-5p inhibitor (i.e., MSC-Exo^anti-let-7a-5p^) or a negative control oligonucleotide (i.e., MSC-Exo^nc^). The result of immunofluorescence staining showed that compared with MSC-Exo^nc^ group, nuclear translocation of TFEB was abolished in MSC-Exo^anti-let-7a-5p^ group (Fig. 5F, G). Meanwhile, the western blot assays demonstrated pretreatment with let-7a-5p inhibitor failed to induce the accumulation of LC3II after application of Leu compared with the negative control oligonucleotide treatment group (Fig. 5H, I). In addition, the knockdown of let-7a-5p in exosomes abolished the hepatoprotective effect of MSC-Exo or MSC-Exo^nc^ (Fig. 5J, K). Taken together, the above data confirmed that MSCs mediate TFEB nuclear translocation and autophagic flux repairment partially via exosomal delivery of let-7a-5p, thereby dampening liver injury in ACLF.

### The effect of let-7a-5p on TFEB nuclear translocation and autophagic flux repairment is mediated by the dephosphorylation of MAP4K3

**Fig. 6:**
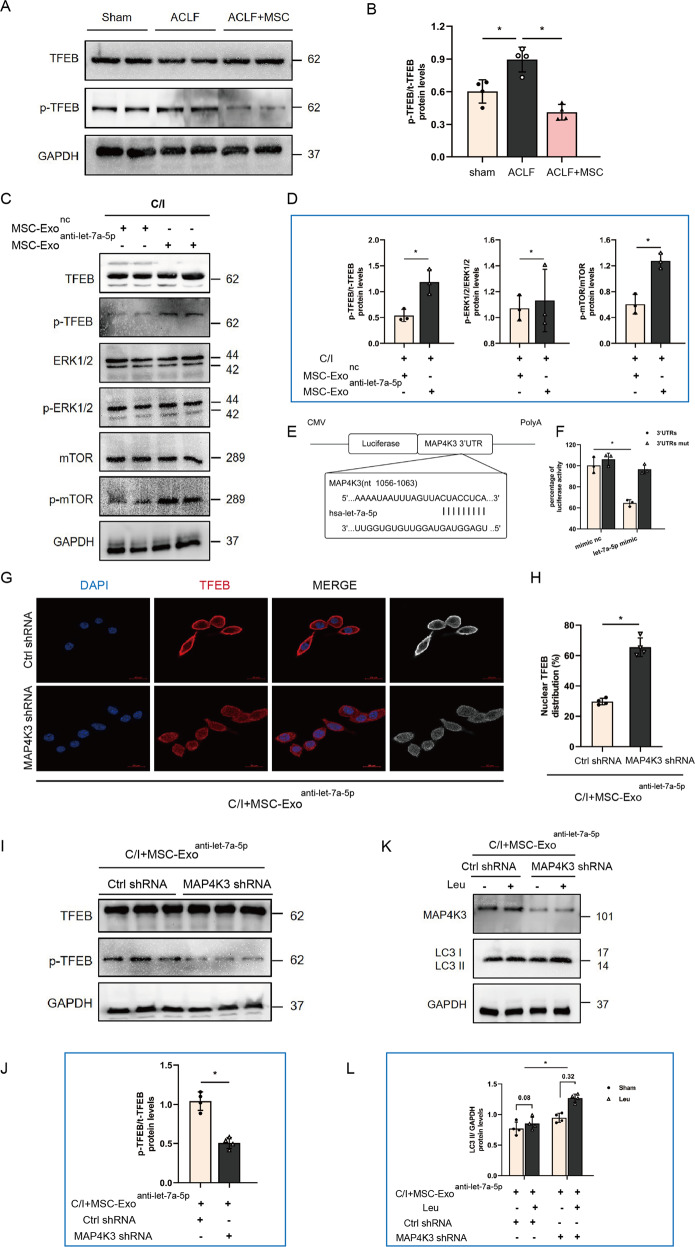
MSC-Exo let-7a-5p and its target MAP4K3 regulate the phosphorylation and subcellular localization of TFEB. **A**, **B** Protein levels of p-TFEB and TFEB (normalized to GAPDH) in hepatocytes from sham group and ACLF mice treated with either vehicle buffer or MSCs were measured at 6 h after transfusion by western blot. Representative images were presented and bar graphs are quantified results of relative gray value in protein bands by Image J (*n* = 4 per group). Data represent mean ± SD, ns nonsignificant, *p < 0.05, one-way ANOVA with Bonferroni’s post analyses. **C**, **D** Obtained MSC-Exo were pretreated with let-7a-5p inhibitor (MSC-Exo^anti-let-7a-5p^) or a negative control oligonucleotide (MSC-Exo^nc^), and then co-cultured with L02 cells bearing 3 h C/I exposure, proteins levels of p-TFEB, TFEB, ERK1/2, p-ERK1/2, mTOR, p-mTOR (normalized to GAPDH) in L02 cells of the above two co-cultured groups were measured by western blot. Representative images were presented and bar graphs are quantified results of relative gray value in protein bands by Image J (*n* = 4 per group). Data represent mean ± SD, ns, nonsignificant, *p < 0.05, student’s test was performed. **E** Schematic illustration of reporter constructs containing the predicted let-7a-5p binding sites in the 3’ UTR of MAP4K3. **F** Relative luciferase activity in L02 cells co-transfected with let-7a-5p-mimic and reporter plasmid constructs containing either the WT or mutated 3′-UTR of MAP4K3 (*n* = 3 per group). Data represent mean ± SD, *p < 0.05, student’s test was performed. **G**, **H** L02 cells were infected with MAP4K3 shRNA or Ctrl shRNA, and then bearing 3 h C/I with MSC-Exo^anti-let-7a-5p^ co-culture. Immunofluorescence staining for TFEB in L02 cells of above two groups was performed to show the subcellular localization of TFEB, representative images were presented (*n* = 4 per group) and red channel fluorescence images which indicated TFEB were converted to white and black and listed in the rightmost column for clearer contrasts of different subcellular compartments. Measurement of nuclear TFEB fluorescence intensity was calculated by percentage of total fluorescence. Red, TFEB; blue, DAPI; ×630 magnification, scale bar, 20 µm. Data represent mean ± SD, *p < 0.05, one-way ANOVA with Bonferroni’s post analyses. **I**, **J** Protein levels of p-TFEB and TFEB (normalized to GAPDH) in L02 cells of above two groups were measured by western blot. Representative images were presented and bar graphs are quantified results of relative gray value in protein bands by Image J (*n* = 4 per group). Data represent mean ± SD, ns, *p < 0.05, student’s *t*-test was performed. **K**, **L** 100 μM leupeptin was added to block lysosomal proteolysis for autophagic flux assay at 4 h before protein extraction. LC3 II protein levels in L02 cells of the above two groups both before and after Leu intervention were measured by western blot. Both of LC3 II protein levels before and after Leu intervention were quantified, and the absolute changes indicating autophagic flux (*n* = 4 per group). Data represent mean ± SD, *p < 0.05. Student’s *t*-test was performed.

To verify the mechanism by which MSC-Exo derived let-7a-5p induced nuclear translocation of TFEB, alteration in signaling pathways correlated with TFEB subcellular localization was investigated. The result of western blot showed the levels of phosphorylated TFEB were significantly lower in ACLF + MSC group mice, which suggested that MSCs could promote the transport of TFEB to the nucleus by dephosphorylation (Fig. 6A, B). Previous studies showed activity of TFEB nuclear translocation was mainly dependent on the levels of TFEB phosphorylation, and the mechanism target of MTOR and ERK are the two main signaling pathways leading to phosphorylation of TFEB [30–33]. Thus, in the present study, we next examined whether the regulation of TFEB nuclear expression by MSC-Exo derived let-7a-5p is related to the phosphorylation of the above pathway. As shown in Fig. 6C, D, concomitant with increased TFEB phosphorylation levels at Ser211, phosphorylated MTOR levels were also dramatically upregulated in groups of cells were treated with MSC-Exo^anti-let-7a-5p^. However, phosphorylated ERK1/2 levels were not significantly different between the groups. Next, the bioinformatic database Targetscan was applied to explore the specific target of let-7a-5p, which may involve in the phosphorylation of MTOR, and we found that 3’ UTR of MAP4K3, which was reported to activate MTOR signaling via promoting the formation of MTORC1 is predicted to be a binding-target sequence of let-7a-5p. Furthermore, to assess whether there was a direct interaction between let-7a-5p and MAP4K3, luciferase reporter plasmid containing either wild-type or mutant 3’-UTRs of MAP4K3 was constructed, and the binding sites of let-7a-5p were shown in Fig. 6E. The result showed that compared with the mutant 3’UTRs construct (3’UTRs mut), administration of let-7a-5p-mimic could significantly reduce the luciferase activity of wide-type MAP4K3 3’UTRs (Fig. 6F). Collectively, the above results implicated the involvement of MAP4K3 in MSC-Exo derived let-7a-5p mediated TFEB nuclear translocation.

To further clarify whether the induction of TFEB nuclear translocation and autophagic flux repairment by MSC-Exo derived let-7a-5p was dependent on MAP4K3 inhibition, the expression of MAP4K3 in groups of cells co-cultured with MSC-Exo^anti-let-7a-5p^ were knockdown by shRNA. The results showed that in the case of let-7a-5p absence in MSC-Exo, knocked down MAP4K3 could rescue the expression of TFEB in the nucleus and reduce phosphorylation levels of TFEB (Fig. 6G through J). Moreover, the magnitude of Leu-induced LC3-II accumulation in cells co-cultured with MSC-Exo^anti-let-7a-5p^ was also significantly larger in MAP4K3 shRNA group than in the control shRNA group (Fig. 6K, L).

**Fig. 7:**
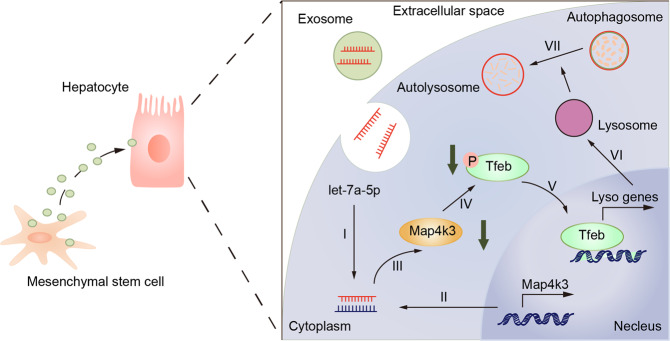
Possible mechanism underlying interaction between MSCs and autophagic flux in ACLF. the pro-autophagic effects of MSCs were partly benefit from its exosomal let-7a-5p targeting MAP4K3, which induce TFEB dephosphorylation and nuclear localization in hepatocytes of ACLF, therefore increasing the transcriptional activity of lysosomal or autophagy-related gene.

All in all, these results showed that MSCs treatment can downregulate MAP4K3 protein kinase expression through secreting let-7a-5p, which was enriched in exosomes, thus inhibiting TFEB phosphorylation and inducing its nuclear translocation, thereby promoting the repairment of autophagic flux and alleviate liver injury in ACLF (Fig. 7).

## Discussion

Due to the lack of suitable experimental models for ACLF and the inaccessibility of liver tissue from ACLF patients, it has remained difficult to analyze the pathophysiological mechanism of ACLF and develop new strategies or improve existing therapies for the disease. In our study, we developed a mouse model of ACLF by chronically intraperitoneal injection of low-dose tetrachloromethane to induce persistent liver injury, followed by an acute sublethal dose of tetrachloromethane injection to initiate an acute burst of liver damage. This model recapitulates the key features of ACLF, which involves chronic liver injury and acute liver insult, moreover, the high short-term mortality, sharply increased serum aminotransferase levels, massive hepatocytes necrosis, and obvious intrahepatic hemorrhage and inflammatory cell infiltration developed in the model were also closely concordant with the histopathological change in patients with ACLF [5]. These results strongly suggest that the model reported in this manuscript represents an experimental setting to further probe the disease mechanisms and therapeutic interventions in ACLF.

Ample lines of evidence have identified MSCs as a promising approach for ACLF due to their distinguishing features on immunomodulatory and hepatoprotective capacities [6–8]. In our previous clinical study, MSCs treatment has been shown to significantly improve the liver function of ACLF patients, improve their survival rate and reduce the incidence of complications such as severe infection, but the fatality rate of patients is still around 30% [8]. Thus, further investigation of molecular mechanisms is essential to optimize the therapeutic potential of MSCs in the treatment of ACLF.

Autophagy is a highly dynamic and multistep process that involves the wrapping of damaged organisms and macromolecules, formation of autophagosomes, fusing of autophagosomes into lysosomes, and degradation of the autolysosomes [25], and evidence has accumulated that changes of autophagic activity can aggravate or attenuate the pathophysiology of liver failure [34–37]. Most recently, increasing reports underlined that a “static” analysis of autophagy cannot provide insights into the autophagic flux [35, 36]. From a dynamical point of view, increased LC3-II levels would be a response to increased autophagosome formation, or a block in autophagosomes fused with lysosomes [38]. Studies in bile duct-ligated (BDL) hepatic fibrosis mice or chronic ethanol-induced (Gao binge) liver injury mice reported that LC3-II is increased, suggestive of induced autophagy, however, the degradative protein p62 was also found accumulated, which points to an impaired autolysosome function [35, 36]. Moreover, hepatocellular carcinoma (HCC) can be induced with the accumulation of p62 via activating Nrf2/c-myc and Wnt/β-Catenin pathway, implying the involvement of insufficient autophagy in the pathogenesis of liver injury and HCC development [39, 40]. Therefore, restoring the autophagy-lysosomal function could be a novel, previously unappreciated therapeutic target. Herein, our in vivo and in vitro data showed an upregulated level of LC3II and p62 in ACLF model, referred to as a blockage of autophago-lysosomal fusion at the end-stage of autophagic flux. However, we also found a number of surprises. MSCs administration not only reduced the inflammatory response and delayed disease progress of ACLF, but also notably decreased the LC3II and p62 levels, which implied that MSCs could restore the pathological impaired autophagy in ACLF and facilitate the degradation of p62. Moreover, aggravated liver damage of ACLF + MSCs mice after phagosome-lysosome fusion impeded by leupeptin highlighted the later clearance stages of autophagosome-lysosome fusion in autophagy and its role in MSCs-mediated hepatic protection.

In recent decades, the MiT/TFE family member, transcription factors EB (TFEB) has been considered a master regulator of lysosome biosynthesis and autophagy-related gene transcription. Once activated, TFEB could promote not only lysosomal-related gene expression under a coordinated lysosomal enhancement and regulation (CLEAR) signal network but also upregulate genes involved in early-stage autophagy [28]. Besides, owning to the effect on autophagy, TFEB was reported to involve in the development and progression of liver disease. Overexpression of TFEB was able to inhibit inflammation and decrease hepatocyte death in ethanol- and copper-induced models of liver injury [36, 37]. In our present study, we observed for the first time that nuclear expression of TFEB was reduced in ACLF hepatocytes, furthermore, MSCs transfusion induced accumulation of TFEB in the nucleus and promoted transcription of autophagy and lysosomal genes, indicating that MSCs-mediated hepatoprotective effects may rely on TFEB-activated lysosome-autophagy pathways. In support of this, in vitro experiment was applied and found that TFEB silencing significantly blocked autophagic flux and reduced lysosome formation in hepatocytes co-cultured with MSCs, and dampened the hepatoprotective role of MSCs with increased cell death. Indeed, further validated whether the lysosome biogenesis activity was involved in MSC-inducing autolysosome formation and if the TFEB control autophagic flux by affecting their expression was proposed and performed in our supplementary experiments. the results of immunohistochemical staining showed a greater and enlarged enrichment of LAMP1 granules in hepatocytes of ACLF group, whereas the granules of CTSB are relatively dispersed (Fig. S2C), which indicated that lysosomal function may be impaired and lysosomal membrane permeabilization (LMP) was promoted in hepatocytes under ACLF conditions. Besides, the immunofluorescence staining of LGALS3, which was highly sensitive to analyze the lysosomal integrity [41], showed that the lysosomal membrane was deficient in hepatocytes of ACLF and MSCs transfusion was implicated in lysosomal membrane repair, as evidenced by LGALS3 puncta were gather in lysosomes and form fluorescent spots in C/I group and a relatively more diffuse distributed and fewer fluorescent spots was founded in cells treated with MSCs (Fig. S2D). Additionally, the higher number of LGALS3 fluorescent spots after TFEB knockdown supported that TFEB favors LMP repair and functions in MSC-inducing autophagosome-lysosome fusion (Fig. S2D). In accordance with this, the specific regulatory mechanism of LMP has been proposed, and further LAMP1 and RAB7 double staining were performed to explore whether the protective effect of TFEB on MSCs-induced autophagy restoration was attributed to lysosome biogenesis and clearance of damaged lysosomes. The results of IF staining confirmed that transfusion of MSCs could promote lysosome synthesis and partly replenish pre-existing dysfunctional lysosomes, as evidenced by an increased number of vesicles for LAMP1 positive alone, which indicated primary lysosomes, in contrast, inhibition of TFEB may impede lysosome biogenesis and reduce the clearance of damaged lysosomes as detected by both of LAMP1- and RAB7-positive vesicles which represented secondary lysosomes were increased appreciably (data not shown). These data elucidated that the effect of MSCs on autophagy rejuvenation was in part ascribed to its ability to active TFEB, maintaining the integrity of the lysosomal membrane and restoring normal function by promoting lysosome biogenesis and clearance of damaged lysosomes. However, since the sophisticated series of membrane phenomena and complex interplay between the constituent players remains poorly understood and the mechanism underlying autophagosome-lysosome fusion is beyond the scope of this manuscript, we tentatively conclude that TFEB accelerated autophagosome-lysosome fusion by promoting the lysosomal assembly of cathepsin and induction of lysosomal acidification, nevertheless the detailed mechanism warrants future studies. Herein, how MSCs promotes nuclear localization of TFEB was addressed. Mechanistically, recent studies have validated that the therapeutic effects of MSCs transplantation on liver tissue repair were partly attributed to its secreted exosomes [14–16]. There, MSC-Exo, which was well described as a potential intercellular communicator that exchanges cellular substances and bioinformation and owned the same roles in the immunoregulatory and pro-regenerative capacity as MSCs, exhibited lower immunogenicity, enhanced homing and prolonged survival, and avoid the risks of replicative senescence, pulmonary embolism, and intractability microenvironment impacts when comparing with MSCs [39]. Here, our data also showed that MSC-Exo play a key role in MSCs-mediated TFEB nuclear translocation of ACLF, as evidenced by decreased nuclear expression of TFEB following application of exosomes inhibitor GW4869. Thus, it represents an interesting therapeutic target, and the concrete regulatory mechanisms for TFEB nuclear translocation still need to be fully elucidated.

To further clarify the exosomal components involved in modulating nuclear import of TFEB, miRNA-Seq analysis in MSC-Exo was performed. Among those highly enriched miRNAs, we noticed that let-7a-5p was lately reported to be markedly reduced in the setting of ACLF and related to 30-day mortality of patients [29]. Besides, let-7 was shown to implicate cell-cycle regulation, cell proliferation, and differentiation. In cholestasis mice, enhancement of let-7 is able to promote liver repair by inhibiting Lin28 expression and facilitating cell functional maturation [42]. Moreover, let-7 was shown to prevent the initiation and development of HCC by repressing the stemness of cancer stem cells and promoting its differentiation [40]. In current data, a lower level of let-7a-5p was also found in liver tissue of ACLF murine, and transfusion of MSCs could induce let-7a-5p expression. Combined with findings that upregulation of let-7 could suppress the expression of inflammatory cytokines and chemokines, including IL-6, IL-1β, IL-8, CCL2, and TNF-α [43–45], it is conceivable to speculate that let-7a-5p could alleviate liver injury by attenuating the uncontrollable cytokine storm in ACLF. To validate this conjecture, we used interfering RNAs and showed that the severity of hepatic impairment and inflammation was significantly exacerbated by let-7a-5p knockdown in vitro. Furthermore, we have also discovered an unexpected and new phenomenon: an increased level of let-7a-5p can be led to cell death from more skewed toward apoptosis rather than necroptosis. As we know, necroptosis has long been considered a trigger for various inflammatory activities, and it presents features that distinguish it from apoptosis in cell morphology and biochemistry, including organelle swelling and rupture; membrane hyperpermeability and integrity disruption; and intercellular proinflammatory factors liberation. This may also partially explain the inflammatory cascade in the condition of ACLF, which exhibit a lower level of let-7a-5p, but the magnitude of the effect and the underlying mechanism remained to be elucidated.

Currently, studies on TFEB posttranscriptional regulation mainly concern phosphorylation by mTOR and MAPK kinases [30–33]. In detail, TFEB is highly phosphorylated at Ser142, Ser211, and Ser3 and retained in the cytoplasm under basal conditions. However, under conditions of elevated stress, cytoplasmic TFEB is dephosphorylated and translocated into the nucleus, resulting in its target gene expression increased. Among that, mTOR complex 1 (mTORC1) phosphorylated TFEB at Ser142 and Ser211, creating a 14-3-3 consensus binding site orientated in cytosol [30, 33]. MAPK family members, ERK2 phosphorylates TFEB at Ser142, whereas JNK and p38 MAPK phosphorylates ZKSCAN3 and inhibit TFEB nuclear translocation [32]. Moreover, Hsu et al. revealed that MAP4K3, another member of MAPK pathway, was able to inactivate TFEB from both directly phosphorylating TFEB at Ser3 or indirectly phosphorylating TFEB at Ser211 and Ser142 by stimulating mTORC1 [31]. In this regard, let-7a-5p was recently found to bind with the MAP4K3 mRNA 3’-UTR and inhibit its translation. Consistent with previous literature [46], our data of dual-luciferase reporter assay determined that MAP4K3 was located in the seed region of a predicted microRNA binding site for let-7a-5p and thus directly alter its expression. In addition, we knock-downed let-7a-5p expression in MSC-Exo by anti-miR and obtained MSC-Exo^anti-let-7a-5p^, and our data provide exquisite evidence that knockdown of let-7a-5p could weaken the effect of MSC-Exo on promoting autophagic flux, accompanied with reduced TFEB nuclear translocation than that of MSC-Exo. Noteworthily, we further validated that inhibition of MAP4K3 expression by let-7a-5p is one important driver for elevated expression of TFEB in the nucleus, as the reduced nuclear presence of TFEB in MSC-Exo^anti-let-7a-5p^ group partially reversed after knocking down MAP4K3 in hepatocytes. The above results indicate that let-7a-5p is a crucial mediator in MSCs-induced TFEB nuclear translocation and autophagic flux restoration. Nevertheless, it should be noted that mechanisms investigated in vitro may not always reflect events in biologically relevant events, the pro-autophagic effect and hepatoprotective role of let-7a-5p and the dynamics change of let-7a-5p and autophagic activation affect disease severity throughout the course of ACLF still need to be validated in further in vivo studies.

In summary, our study demonstrated for the first time that MSCs could promote autophago-lysosomal fusion at the end-stage of autophagic flux and thus restrict inflammation and alleviate liver injury in the model of ACLF. Mechanistically, we show that the pro-autophagic effects of MSCs were partly beneficial from its exosomal let-7a-5p to induce nuclear localization of TFEB by targeting MAP4K3. These results open a novel insight into the functional linkage between autolysosome maturation and liver inflammation and suggest that let-7a-5p could be a potential target in the treatment of ACLF.

## Supplementary information

Figure S1

Figure S2

supplementary table 1

supplementary table 2

supplementary materials and mathods

supplementary figure legend

full and uncropped western blots

Reproducibility Checklist

## Data Availability

All data generated or analyzed during this study are included in this published article and in its supplementary information file.
