# Supplementary figures and images for: Mesenchymal stem cells exosomal let-7a-5p improve autophagic flux and alleviate liver injury in acute-on-chronic liver failure by promoting nuclear expression of TFEB
Source: Cell Death Dis. 2022 Oct 12;13(10):865. doi: 10.1038/s41419-022-05303-9 (PMC9556718; doi:10.1038/s41419-022-05303-9)

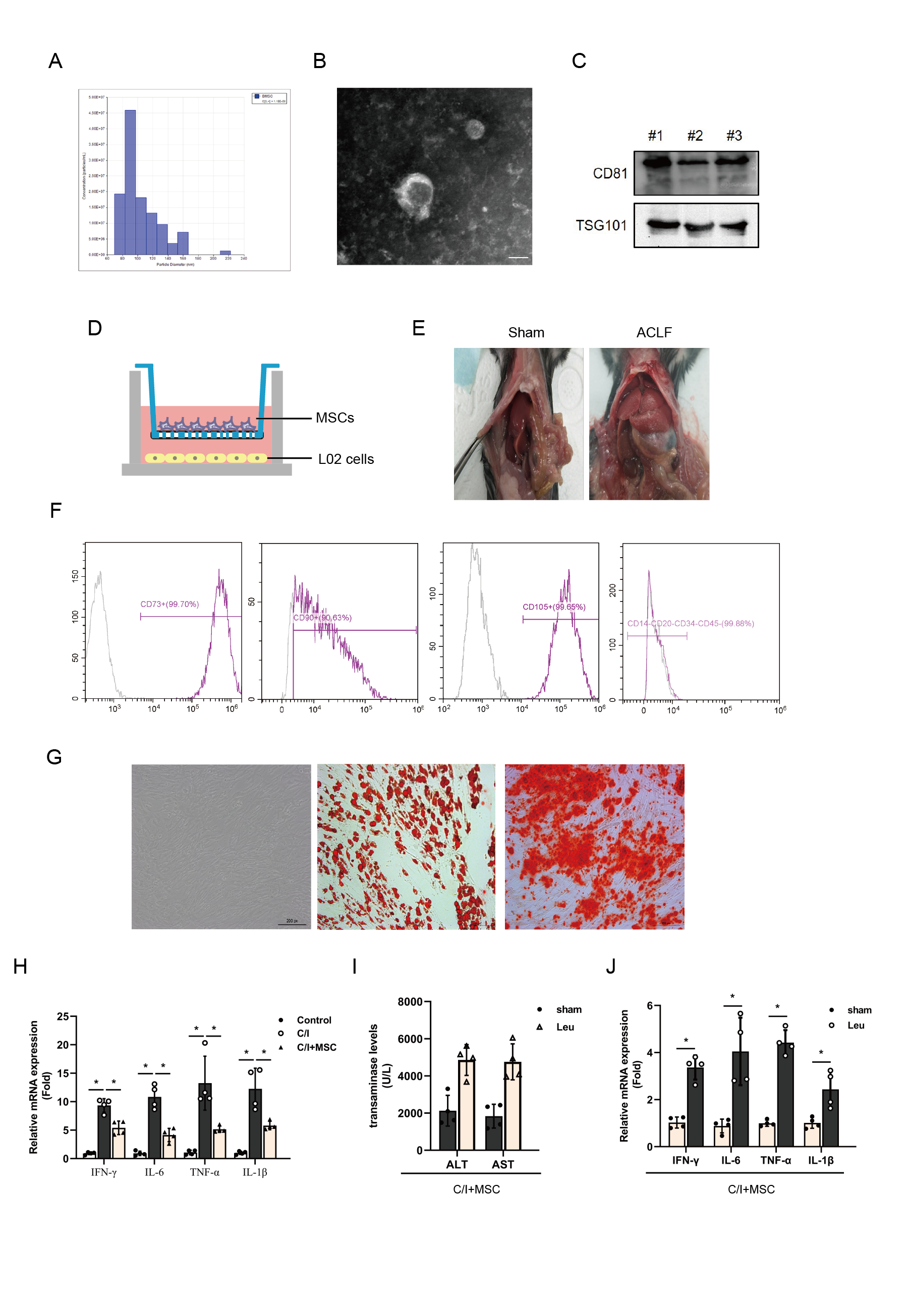

Supplement: Supplementary file 1 — Figure S1 [file 41419_2022_5303_MOESM1_ESM.png]

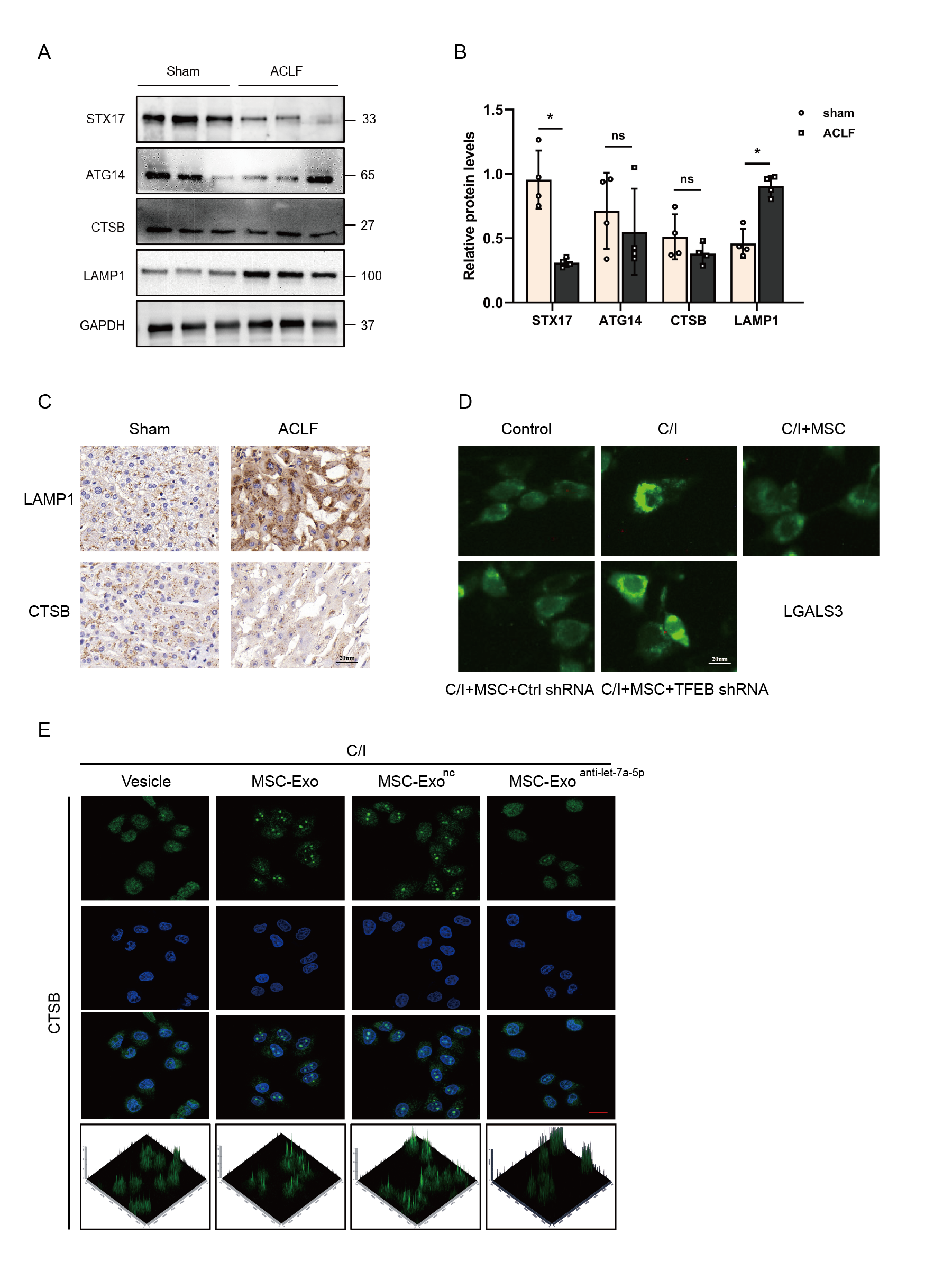

Supplement: Supplementary file 2 — Figure S2 [file 41419_2022_5303_MOESM2_ESM.png]
