# Supplementary material for: Mesenchymal stem cells exosomal let-7a-5p improve autophagic flux and alleviate liver injury in acute-on-chronic liver failure by promoting nuclear expression of TFEB
Source: Cell Death Dis. 2022 Oct 12;13(10):865. doi: 10.1038/s41419-022-05303-9 (PMC9556718; doi:10.1038/s41419-022-05303-9)
Supplement: Supplementary file 3 — supplementary table 1 [file 41419_2022_5303_MOESM3_ESM.docx]

**Table S1**

**qRT-PCR Primers:**

| **Gene** | **Species** | **Primer** | **Sequence** |
| --- | --- | --- | --- |
| *IFN-γ* | Mouse | Forward | 5’-CTTGAAAGACAATCAGGCCATC-3’ |
|  |  | Reverse | 5’-CTTGGCAATACTCATGAATGCA-3’ |
| IL-6 | Mouse | Forward | 5’-CTCCCAACAGACCTGTCTATAC-3’ |
|  |  | Reverse | 5’-CTCCCAACAGACCTGTCTATAC-3’ |
| TNF-α | Mouse | Forward | 5’-ATGTCTCAGCCTCTTCTCATTC-3’ |
|  |  | Reverse | 5’-GCTTGTCACTCGAATTTTGAGA-3’ |
| IL-1β | Mouse | Forward | 5’-CACTACAGGCTCCGAGATGAACAAC-3’ |
|  |  | Reverse | 5’-TGTCGTTGCTTGGTTCTCCTTGTAC-3’ |
| *IFN-γ* | Human | Forward | 5’-GAGATGACTTCGAAAAGCTGAC-3’ |
|  |  | Reverse | 5’-CCTTTTTCGCTTCCCTGTTTTA-3’ |
| IL-6 | Human | Forward | 5’-CACTGGTCTTTTGGAGTTTGAG-3’ |
|  |  | Reverse | 5’-GGACTTTTGTACTCATCTGCAC-3’ |
| TNF-α | Human | Forward | 5’-CGTGGAGCTGGCCGAGGAG-3’ |
|  |  | Reverse | 5’-AGGAAGGAGAAGAGGCTGAGGAAC-3’ |
| IL-1β | Human | Forward | 5’-GCCAGTGAAATGATGGCTTATT-3’ |
|  |  | Reverse | 5’- AGGAGCACTTCATCTGTTTAGG-3’ |
| LAMP1 | Mouse | Forward | 5’-AACAACGGAACCTGCCTGCTG-5’ |
|  |  | Reverse | 5’-TGATGTTGAACGCTCTGGTCACC-3’ |
| TFEB | Mouse | Forward | 5’-CACAGGTTACCCCGATACC-3’ |
|  |  | Reverse | 5’-AGGGAGTCATCTAGGAGCATTA-3’ |
| CTSD | Mouse | Forward | 5’-CATCTATCCGTCGGACTATGAC-3’ |
|  |  | Reverse | 5’-ATCAAAGACGACTGTGAAACAC-3’ |
| CTSB | Mouse | Forward | 5’-CTCATGTAGGCTGCTTACCATA-3’ |
|  |  | Reverse | 5’-CTCATGTAGGCTGCTTACCATA-3’ |
| MAP1LC3B | Mouse | Forward | 5’-CCAGCAGTGTTGTCAGCAGGTC-3’ |
|  |  | Reverse | 5’-TGTCATTCGTGGCTGTCAAGGTTC-3’ |
| BECN1 | Mouse | Forward | 5’-GGACCAGGAGGAAGCTCAGTACC-3’ |
|  |  | Reverse | 5’-CGCTGTGCCAGATGTGGAAGG-3’ |
| ATG5 | Mouse | Forward | 5’-GCAAGCCAAGGAGGAGAAGATTCC-3’ |
|  |  | Reverse | 5’-GTGTCTCAGCGAAGCAGTGGTG-3’ |
| SQTSM1 | Mouse | Forward | 5’-AGGAGGAGACGATGACTGGACAC-3’ |
|  |  | Reverse | 5’-TTGGTCTGTAGGAGCCTGGTGAG-3’ |
