# Supplementary material for: Mesenchymal stem cells exosomal let-7a-5p improve autophagic flux and alleviate liver injury in acute-on-chronic liver failure by promoting nuclear expression of TFEB
Source: Cell Death Dis. 2022 Oct 12;13(10):865. doi: 10.1038/s41419-022-05303-9 (PMC9556718; doi:10.1038/s41419-022-05303-9)
Supplement: Supplementary file 5 — supplementary materials and mathods [file 41419_2022_5303_MOESM5_ESM.docx]

***Supplementary materials and methods***

***Characterisation of MSCs***

After 3th passaged of Mesenchymal stem cell (MSC), the expression of surface markers CD73, CD90, CD105 and hematopoietic markers CD14, CD20, CD34 and CD45 were detected with MSC phenotyping kit (#130-095-198, Miltenyi Biotec, Germany) according to the manufacturer’s instructions. Briefly, MSC was digested and collected after attaining 70–80% confluence, followed by washing with 1% BSA (#1933, Sigma, USA) and incubating with CD90-FITC, CD105-PE, CD73-APC, CD14/CD20/CD34/CD45-PerCP5.5 and the corresponding isotype control antibodies for 30min (ref. 1). The flow cytometry was detected by CytoFLEX S (Beckman Coulter, USA), and the data were analyzed using CytExpert (Beckman Coulter, USA). At the 5th passage, the multipotential differentiation of MSC was determined by using osteogenesis and adipogenesis medium (#7531-b, #7541-b, ScienCell, USA) (ref. 1). MSC were inoculated in a 6-well plate, and cultured with the above medium respectively. The medium was replaced every 2-3 days. After 21 days, the cells were fixed by 4% paraformaldehyde, and Alizarin Red S or Oil red O working solution (#0223, #0843, ScienCell, USA) were added to each plate for 30 min. The staining was visualized and photographed by a fluorescence microscope (Leica DMI4000B).

***Characterisation of exosomes***

Isolated exosomes were characterised by measuring the expression of the EV-associated protein markers CD81 (#ab109201, Abcam, UK) and TSG101(#ab125011, Abcam, UK) by western blot. The particle size and concentration of exosomes were determined by nanoparticle tracking analysis (Nanosight; UK). The ultrastructure of exosomes was visualized and photographed by transmission electron microscope (TEM; JEM-1400; Japan) (ref. 2).

***Serum ALT/AST test***

Mouse blood samples were collected from tail veins were centrifuged at 2000 rpm/min for 5min. The levels of ALT and AST in the supernatants were detected by automated chemical analyzer (Chemray 240, Rayto Corporation, Shenzhen, China) with the standard test kit (Bestbio, China).

***Histopathological analysis of liver tissue and Immunohistochemistry (IHC)***

After the liver tissues from different groups of mice were fixed and embedded in paraffin, sections which affixed to slides and de-paraffinized were then applied to Hematoxylin and eosin (H&E) staining (#C0105, Beyotime, China) to define the liver morphological changes. For immunohistochemistry (IHC) staining, 4μm-thick sections affixed to slides were deparaffinized with xylene, rehydrated, and then successively subjected to antigen retrieval, followed by quenching of the endogenous peroxidase with hydrogen peroxide. After nonspecific blocking by using goat serum, sections were incubated with primary antibody against LC3 (#PM036, MBL, Japan) and p62 (#PM045, MBL, Japan) overnight at 4 °C and secondary antibody at room temperature for 1h. DAB and Mayer’s hematoxylin solution were applied in final. Each section was observed and photographed under a ﬂuorescent inverted microscope (Leica, Germany). The images were assessed in a blinded fashion and processed and analyzed by using Image J (NIH, USA).

**Transmission electron microscopy (TEM)**

To monitor the formation of autophagosomes and autolysosomes, 1mm3 sized liver tissues from different groups were fixed in 2.5% glutaraldehyde and dehydrated with ethanol and acetone. After embedding and cutting with ultramicrotome (RMC MT6000-XL, USA), 60-80nm ultrathin sections were stained with uranyl acetate and lead citrate solution and then observed under a transmission electron microscope (HT7700, HITACHI, Japan).

***Primary hepatocytes isolation and purification***

Mouse liver from different groups was first perfused with perfusate via the inferior vena cava (IVC) till the effluent fluid without color (3–4 min), followed by 0.25% collagenase IV (#LS004188, Worthington, USA) digestion at a rate of 3ml/min to destroy the connective tissue in the liver (2-3 min). Stopped perfusion when the liver surface appears speckle erosion and gently vibrated the liver with forceps to disperse the cells at 4 °C. Passed the cell suspension through 100μm strainer and centrifuged at 50g for 3min at 4 °C, the sediments were washed and resuspended once in Buffer (without collagenase) and centrifuged at 50 × g for 5 min at 4 °C. Cell pellets were then mixed with 90% Percoll (#17-0891-09, GE Healthcare, USA) and centrifuged at 100 × g for 10 min. were then purified by 90% Percoll (#17-0891-09, GE Healthcare, USA) and washed again in Buffer (without collagenase).

***Gene silencing with shRNA and miRNA antagomirs transfection***

The lentivirus expressing short hairpin RNA (shRNA) against TFEB and MAP4K3 were obtained from Hanheng Biological (Shanghai, China), and lentiviral vector tandem-labeled mCherry-eGFP-LC3 reporter were constructed by GeneChem (Shanghai, China). The transfection was implemented according to the manufacturer’s specification respectively. Briefly, L02 cells were seeded in 12-well plates and cultured with complete medium containing HiTransG P, then transfected with viral fluid at an MOI of 30 when the cells grew to a 30% fusion degree and selected the stable cells by continuously using puromycin for 4-6 weeks. To performed miRNA antagomirs transfection, MSCs was plated in 24-well and cultured in medium without penicillin-streptomycin. has-let-7a-5p inhibitor and inhibitor nc (RiboBio, China) was diluted in OPTI-MEM I medium (#31985070, Gibco, USA) to a working concentration of 50nM and transfected into MSCs with LipofectamineTM 2000 reagent (#11668019, ThermoFisher, USA). After 24 h, the medium was replaced with complete medium and cultured to 80% confluency and 24-36 h before exosomes were extracted, the medium was changed to serum-free medium.

***Plasmid construction and luciferase reporter assays***

To generate the luciferase constructs, wild type and mutated putative binding sites of let-7a-5p on MAP4K3 3’-UTR were amplified by PCR and cloned into the pmirGLO Dual-Luciferase miRNA Target Expression Vector (Promega, USA) between the SacI and XbaI. After that, the luciferase constructs were co-transfected into L02 cells with either 30 nM let-7a-5p mimic (#4464066, Ambion, USA) or mimic negative control (#4464058, Ambion, USA) by Lipofectamine 2000 reagents (#11668019, ThermoFisher, USA). At 24 h post transfection, the relative luciferase activity was detected by using the dual-luciferase reporter assay system (Promega, USA) according to the manufacturer’s instruction, the luciferase activity was normalized to endogenous firefly luciferase, and the experiments were repeated three times independently and each group was performed in triplicate wells for each condition.

***Immunofluorescence (IF) and confocal microscopy***

To carry out the immunofluorescence (IF) staining, L02 cells were fixed with 4% paraformaldehyde and permeabilized in 0.2% Triton X-100 for 10min. Then blocked with 5% BSA and incubated with primary antibody against TFEB (#A7311, Abclonal, China) overnight at 4°C, the Alexa Fluor® 594 secondary antibody (#A23420, Abbkine, China) was added and incubated in the dark for 1h. Finally, the nucleus was staining with DAPI (#C1005, Beyotime, China) at room temperature for 5min. The stained cells were visualized under confocal microscope (Zeiss, LSM710, Germany) and photographed, at least 5 randomly selected fields cells were counted in per group. The Cells grown on glass coverslips which pre-transfected with mCherry-EGFP-LC3 dual luciferase lentivirus (Genechem Incorporation, Shanghai, China) and received the designated treatments were fixed with 4% formaldehyde for 30 min and then photographed using a confocal microscope (Zeiss, LSM710, Germany). The images were assessed in a blinded fashion.

***Reverse transcription and quantitative PCR***

Total RNA was isolated from primary hepatocytes and L02 cells by using Trizol reagent (#10296028, Invitrogen, USA) and DNA originally existed was removed with gDNA Eraser (#RR047A, #638315, Takara, Japan) according to the manufacturer’s protocol. After determining the quality and concentration of the extracted RNA, appropriate amount of RNA was reverse transcribed into cDNA by the PrimeScript RT reagent Kit with gDNA Eraser (#RR047A, Takara, Japan) and the remaining RNA was used for reverse transcription of miRNA by Mir-X™ miRNA First Strand Synthesis Kit (#638315, Takara, Japan). For the measurement of mRNA and miRNA which used polyA tailing method for reverse-transcription, Quantitative PCR was performed with TB Green Premix Ex Taq II (Tli RNaseH Plus) (#RR820A, Takara, Japan) in a LightCycler480 System (Roche, USA). Results of mRNA and miRNA were normalized to GAPDH and U6 respectively. The details of primer sequences were provided in supplementary table 1.

***Separation of the protein and western blotting***

Total proteins of primary hepatocytes and L02 cells were isolated by using cold radioimmunoprecipitation assay (RIPA) buffer containing protease and phosphatase inhibitor cocktail (#P1046, Beyotime, China) lysed for 30-60min, and the different component proteins of primary hepatocytes were separated by using nuclear & cytoplasmic protein extraction kit (#P0027, Beyotime, China) according to the manufacturer's instruction. In brief, washed the hepatocytes with phosphate buffer saline (PBS), and then centrifugated at 500 g for 5 min. the pellet was added with cytoplasmic protein extraction reagent A with PMSF, vortexed for 5-10 s to fully suspend and disperse the cell pellet, and incubated on ice for 10-15 min. After 10 uL cytoplasmic protein extraction reagent B was added, the suspended and dispersed cell pellet was vortexed at maximum speed for 5 s and incubated on ice for 1 min. Vortexed at maximum speed for 5 s again and then centrifugated at 16,000g for 5 min. After that, the supernatant was collected as the extracted cytoplasmic protein. For the insoluble fraction, the residual supernatant was completely aspirated and 50 uL nuclear protein extraction reagent with PMSF was added. Vortexed at maximum speed for 15-30 s to fully suspended and dispersed the insoluble fraction. Then placed on ice and vortexed for 15-30 s every 1-2 min, a total of 30 min. Finally, centrifuged at 16,000g for 10 min, and the supernatant was harvested as the extracted nuclear protein. After extraction and quantification of the proteins, equal proteins from different groups were consecutive subjected to 12% SDS-PAGE, transmembrane to the 0.22/0.45um polyvinylidene difluoride (PVDF) (#ISEQ00010, #IPVH00010, Millipore) and incubated with the primary antibodies overnight at 4°C. The Primary antibodies targeting LC3 (#PM036, 1:1000, MBL, Japan), p62 (#PM045, 1:1000, MBL, Japan), TFEB (#A7311, 1:1000, Abclonal, China), Phospho-TFEB (#37681, 1:1000, CST, USA), ERK1/2 (#9102, 1:1000, CST, USA), Phospho- ERK1/2 (#9106, 1:1000, CST, USA), mTOR (##2983, 1:1000, CST, USA), Phospho- mTOR (#5536, 1:1000, CST, USA), MAP4K3 (#92427, 1:1000, Cell Signaling Technology, USA), GAPDH (#AC002, 1:1000, Abclonal, China), Histone H3 (#A2348, 1:1000, Abclonal, China) were used. Then, the blots were incubated with the species-specific HRP-conjugated secondary antibodies and detected under a Gel Imager System (FluorChem M, USA) by using Omni-ECL™ ultra-sensitive chemiluminescence detection kit (#SQ201, EpiZyme, China). Quantification of protein bands were analyzed with Image J.

***Hepatocellular death assay***

To perform TUNEL assay, DeadEnd™ Fluorometric TUNEL System (#G3250, Promega, USA) was applied according to the manual. In brief, the re-fixed liver sections and permeabilized L02 cells were incubated with rTDT buffer for 60min in the dark at 37°C followed by infiltration with SSC solution to stop reaction. After DAPI nuclei staining, the sections were observed and photographed by a fluorescence microscope (Leica DMI4000B, Germany), and at least 5 fields picked in a blind manner were used for assessment of the cell death.

For Annexin/V-PI assay, L02 cells were stained with Annexin/V-PI apoptosis kit (#70-APCC101, MultiSciences, China) by flow cytometry according to the protocol. Briefly, L02 cells were digested by accutase (#70-AT001, MultiSciences, China) and stained with Annexin/V-FITC and PI in 1×binding buffer for 15min at room temperature avoid light irradiation. Percentage of cell death was evaluated under the Cytoflex S (Beckman Coulter, USA), Annexin V+/PI- indicated apoptotic cells while Annexin V+/PI+ suggested necrosis.

**Reference**

[1] Uccelli A, Moretta L, Pistoia V. Mesenchymal stem cells in health and disease. Nat Rev Immunol 8, 726-36 (2008)

[2] Théry C, Witwer KW, Aikawa E, Alcaraz MJ, Anderson JD, Andriantsitohaina R, et al. Minimal information for studies of extracellular vesicles 2018 (MISEV2018): a position statement of the International Society for Extracellular Vesicles and update of the MISEV2014 guidelines. J Extracell Vesicles 7, 1535750 (2018)
