# Supplementary material for: Mesenchymal stem cells exosomal let-7a-5p improve autophagic flux and alleviate liver injury in acute-on-chronic liver failure by promoting nuclear expression of TFEB
Source: Cell Death Dis. 2022 Oct 12;13(10):865. doi: 10.1038/s41419-022-05303-9 (PMC9556718; doi:10.1038/s41419-022-05303-9)
Supplement: Supplementary file 6 — supplementary figure legend [file 41419_2022_5303_MOESM6_ESM.docx]

**supplementary figure 1**

**Figure S1**

**A, B, C)** Nanoparticle tracking analysis, spheroid morphology and size under scanning electron microscope (SEM) and proteins levels of vesicle markers CD81 and TSG101were investigated. **D)** schematic diagram of in vitro co-culture experiments with 3-h C/I exposed L02 cells and MSCs. **E)** macroscopic views of liver tissues in sham and ACLF mice. **F, G)** flow staining of the MSC surface markers CD73, CD90, CD105 and hematopoietic markers CD14, CD20, CD34 and CD45 and the corresponding isotype control antibodies. Oil red O and Alizarin Red S staining in MSCs cultured in osteogenesis and adipogenesis medium to determine the multipotential differentiation of MSCs. **H)** mRNA levels of IFN-γ, IL-6, TNF-α, IL-1β (normalized to GAPDH) in L02 cells of control group and 3-h C/I exposed L02 cells with or without MSCs by qRT-PCR. (n=4 per group). Data represent mean ± SD, *p < 0.05, one-way ANOVA with Bonferroni’s post analyses. **I)** serum transaminases ALT and AST levels in supernatants of L02 cells bearing 3-h C/I with MSCs coculture challenged with or without Leu were determined. (n=4 per group). Data represent mean ± SD, *p < 0.05, student’t test was performed. **J)** mRNA levels of IFN-γ, IL-6, TNF-α, IL-1β (normalized to GAPDH) of above two groups were determined by qRT-PCR. (n=4 per group). Data represent mean ± SD, *p < 0.05, student’t test was performed.

**Figure S2**

**A, B)** Protein levels of STX17, ATG14, CTSB and LAMP1 (normalized to GAPDH) in hepatocytes of sham and ACLF mice were measured by western blot. Representative images were presented and bar graphs are quantified results of relative grey value in protein bands by Image J (n=4 per group). Data represent mean ± SD, ns, non-significant, *p < 0.05, student’t test was performed. **C**) Immunohistochemical staining for LAMP1 and CTSB in liver sections from SHAM and ACLF mice. Representative images were presented from n=4 mice per group. 400× magnification, scale bar, 20 µm. **D)** L02 cells were stably infected with TFEB shRNA or Ctrl shRNA, and then bearing 3-h C/I with MSCs coculture. Immunofluorescent staining of LGALS3 in control group L02 cells, 3-h C/I exposed L02 cells with or without MSCs and the above two cocultured groups was performed, representative images were presented. 200× magnification, scale bar, 50 µm. **E)** Obtained MSC-Exo were pretreated with let-7a-5p inhibitor (MSC-Exo^anti-let-7a-5p^) or a negative control oligonucleotide (MSC-Exo^nc^), and then the obtained MSC-Exo, MSC-Exo^nc^ and MSC-Exo^anti-let-7a-5p^ were cocultured with L02 cells bearing 3-h C/I exposure, immunofluorescence staining for CTSB in L02 cells bearing 3-h C/I exposure alone and the above three cocultured groups was performed and representative images were presented. 2.5D intensity histograms of CTSB were listed at the bottom row for showing the intensity of each punctum. 630× magnification, scale bar, 20 µm.
