# Supplementary material for: Mesenchymal stem cells exosomal let-7a-5p improve autophagic flux and alleviate liver injury in acute-on-chronic liver failure by promoting nuclear expression of TFEB
Source: Cell Death Dis. 2022 Oct 12;13(10):865. doi: 10.1038/s41419-022-05303-9 (PMC9556718; doi:10.1038/s41419-022-05303-9)

Supplemental Figure 3: Uncropped western blot images

Supplemental Figure S3A related to Figure 2C

LC3 17KDa, 14KDa

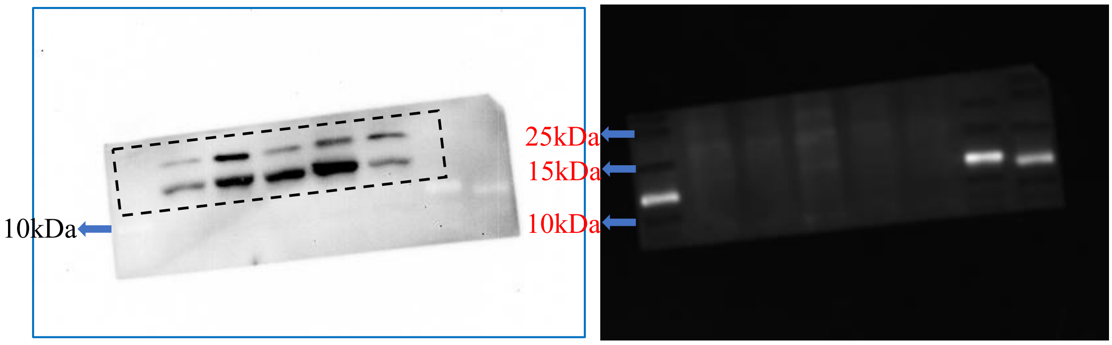

P62 62KDa

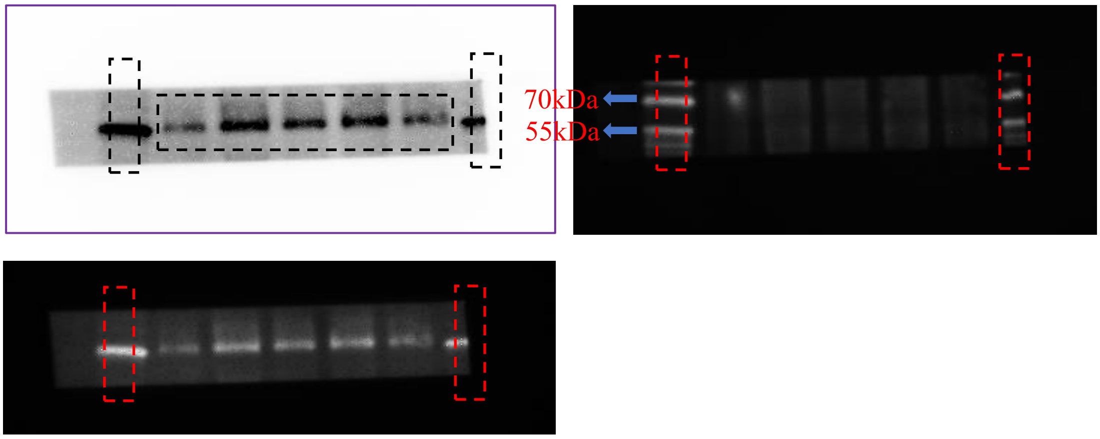

GAPDH 37KDa

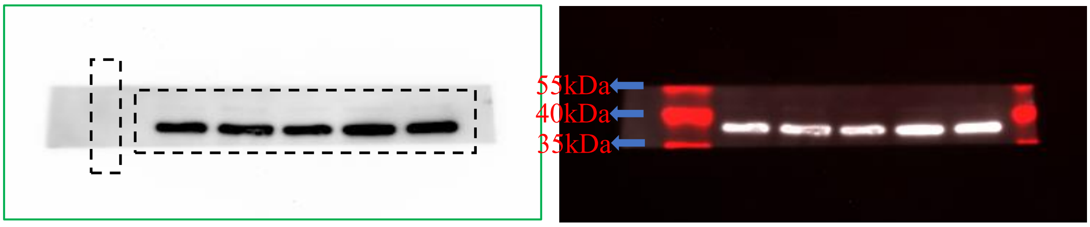

Supplemental Figure S3A related to Figure 2G

LC3 17KDa, 14KDa

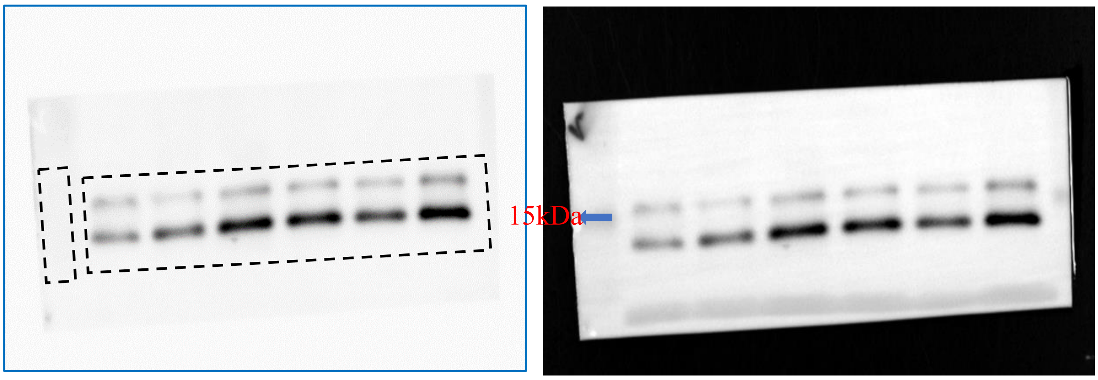

GAPDH 37KDa

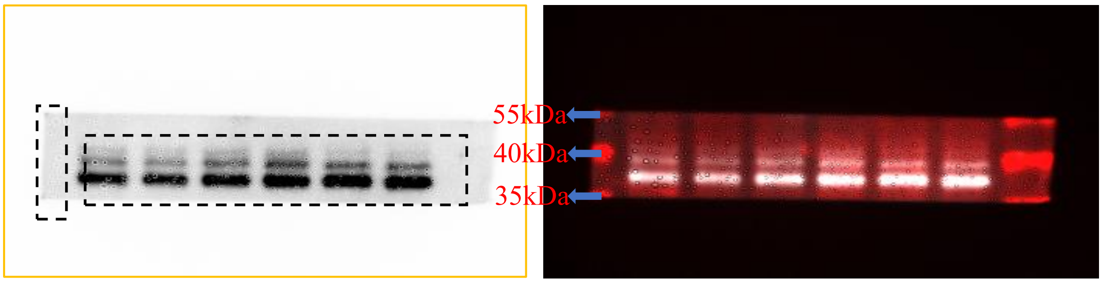

Supplemental Figure S3B related to Figure 4A

Cyt.TFEB 65KDa

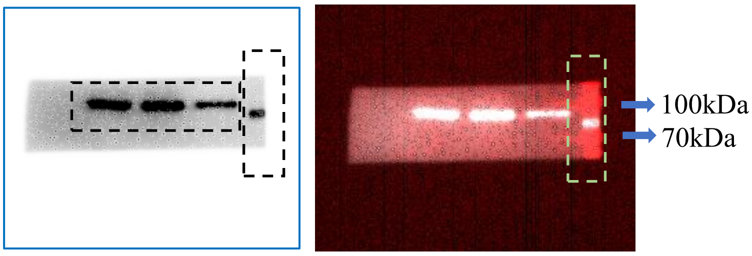

GAPDH 37KDa

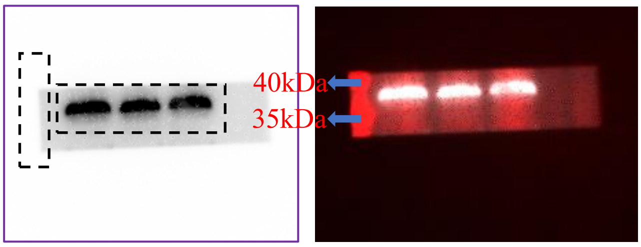

Nucl.TFEB 65KDa

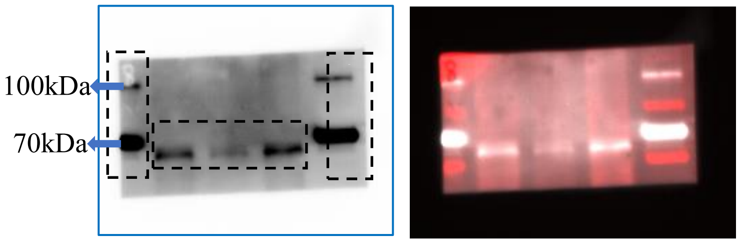

H3 17KDa

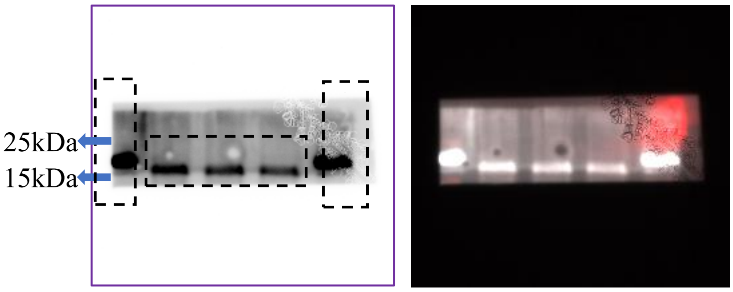

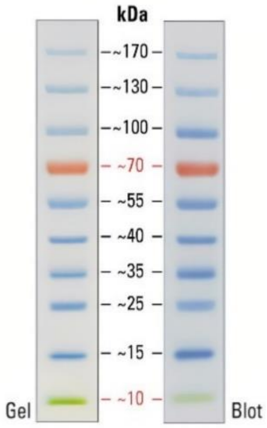

Supplemental Figure S3B related to Figure 4F

LC3 17KDa, 14KDa

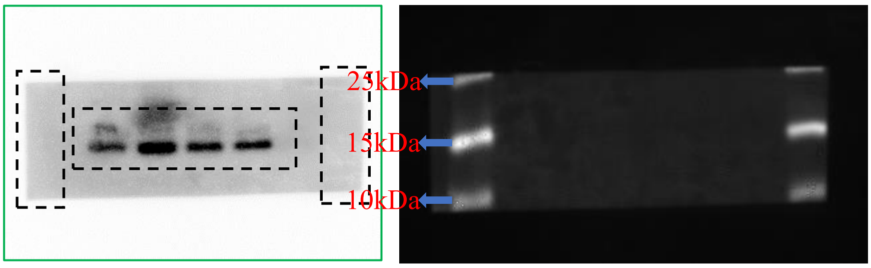

GAPDH 37KDa

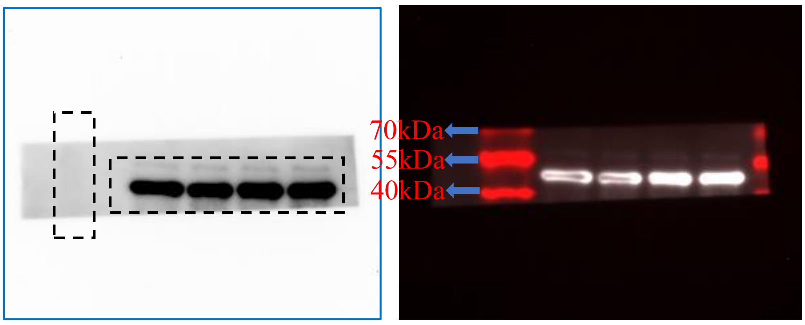

Supplemental Figure S3C related to Figure 5H

LC3 17KDa, 14KDa

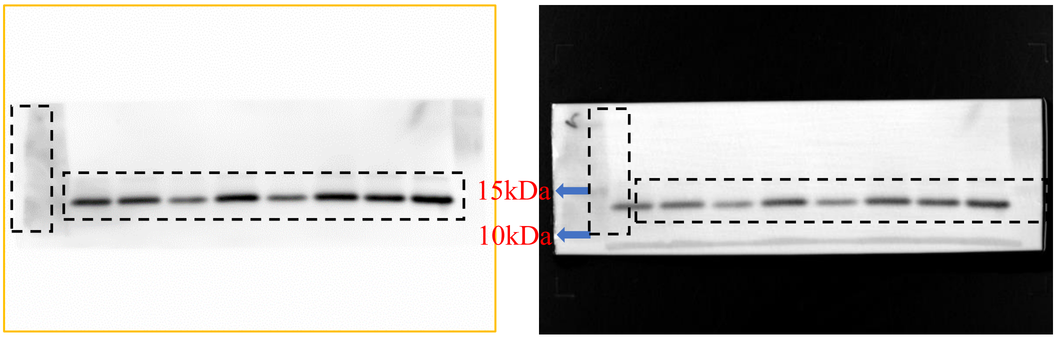

GAPDH 37KDa

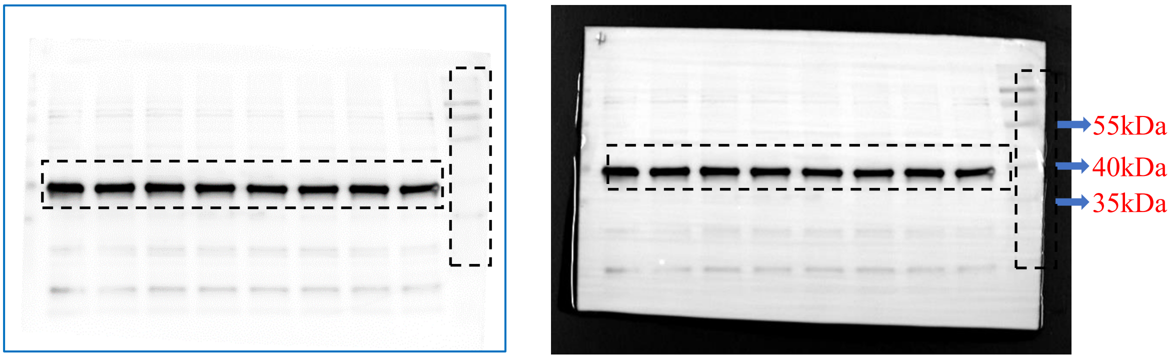

Supplemental Figure S3D related to Figure 6A

TFEB 62KDa

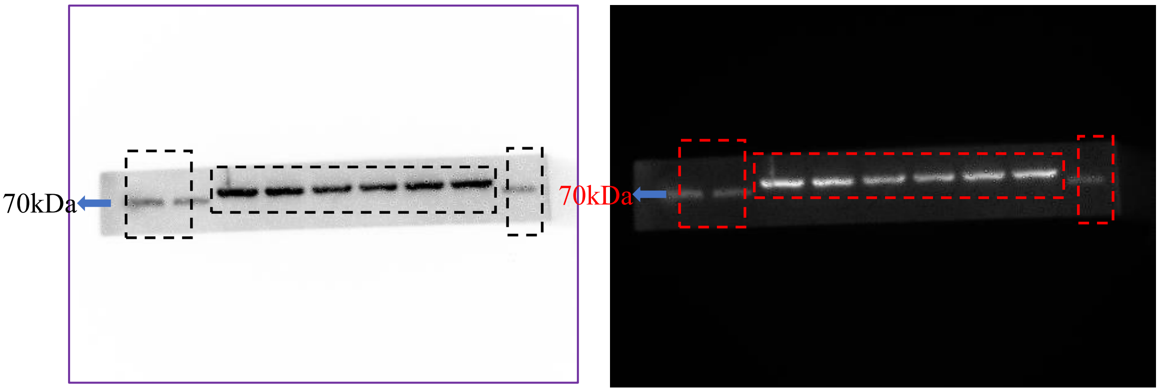

p-TFEB 62KDa

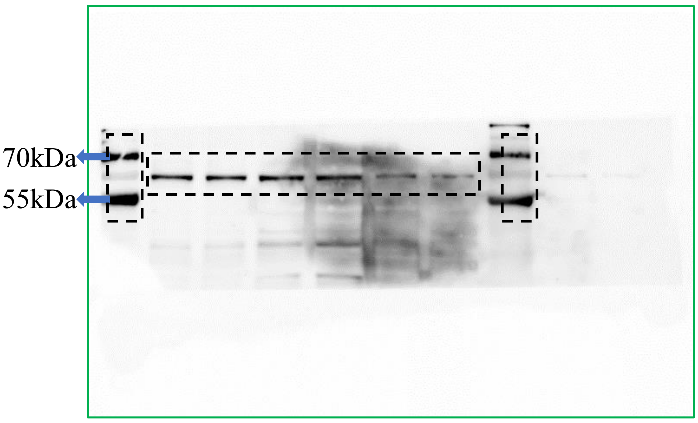

GAPDH 37KDa

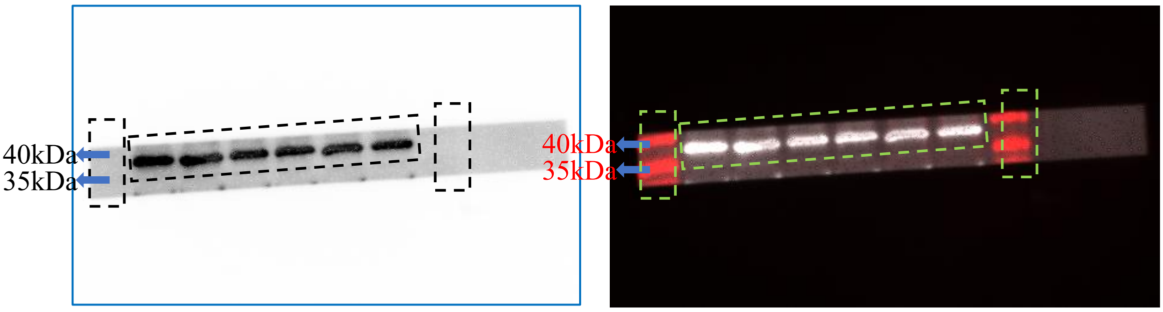

Supplemental Figure S3D related to Figure 6C

TFEB 62KDa

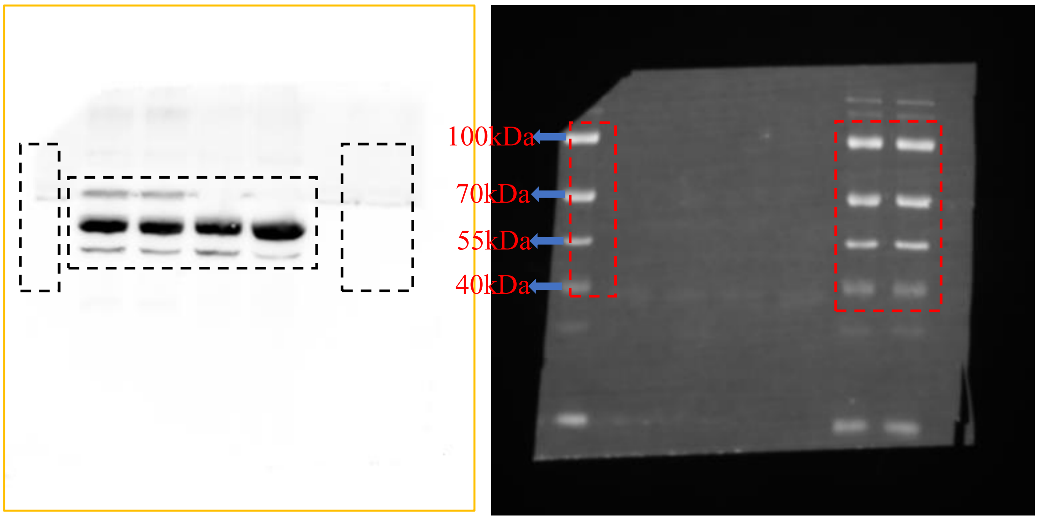

p-TFEB 62KDa

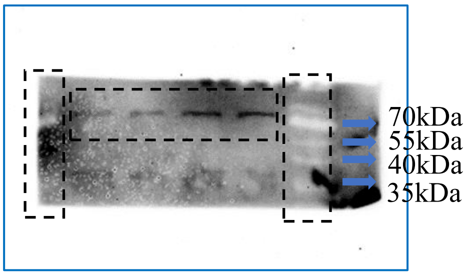

ERK1/2 44KDa, 42KDa

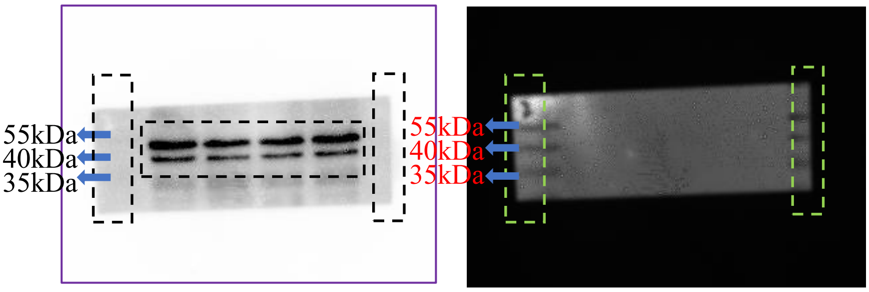

p-ERK1/2 44KDa, 42KDa

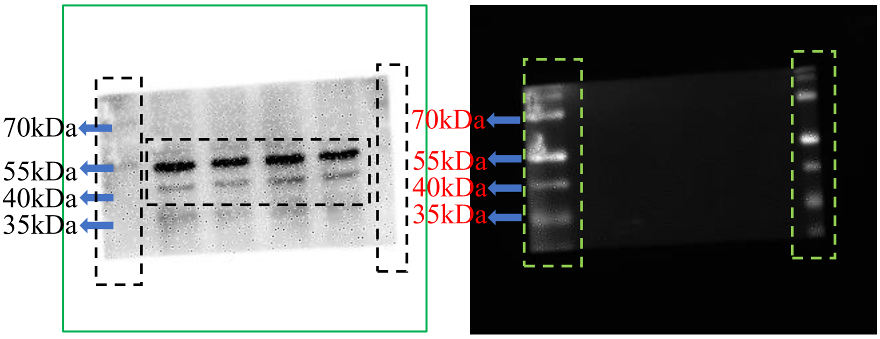

mTOR 289KDa

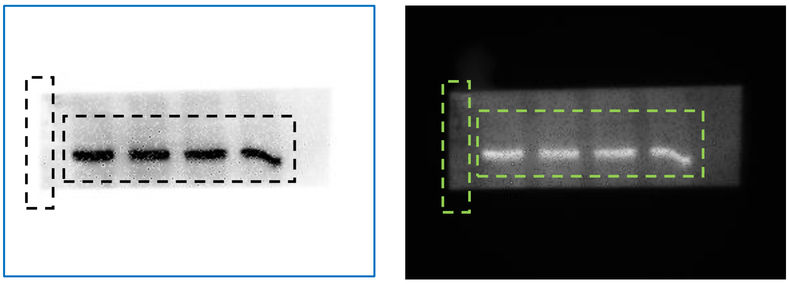

p-mTOR 289KDa

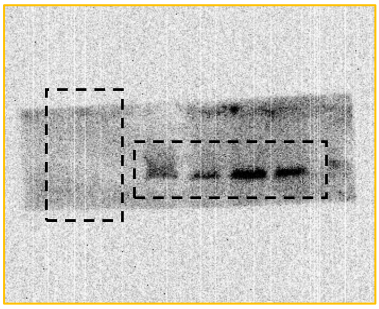

GAPDH 37KDa

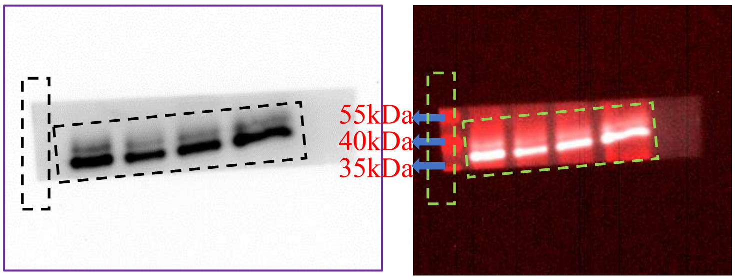

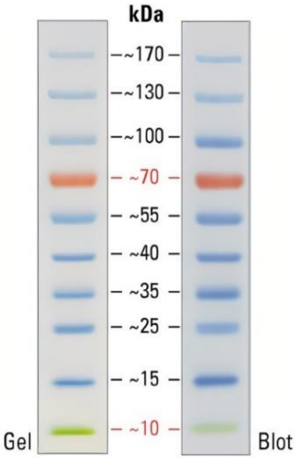

Supplemental Figure S3D related to Figure 6I

TFEB 62KDa

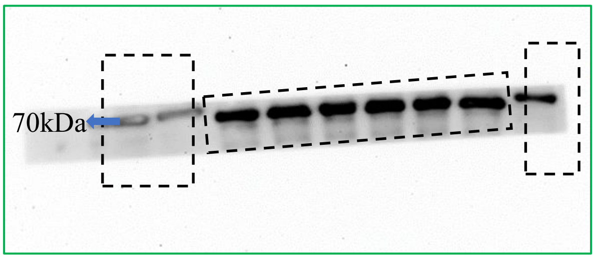

p-TFEB 62KDa

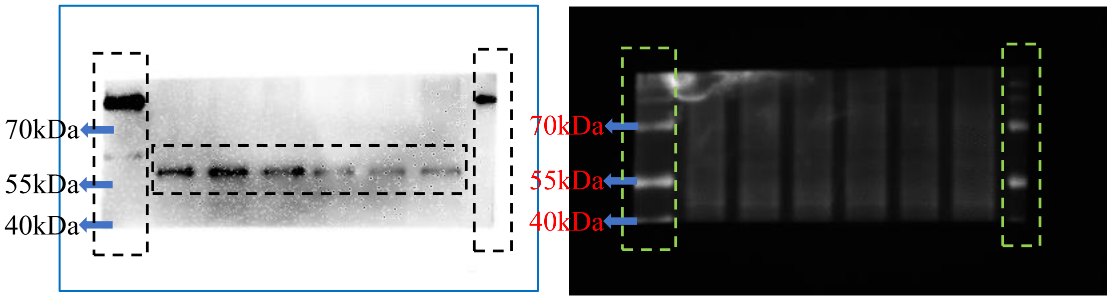

GAPDH 37KDa

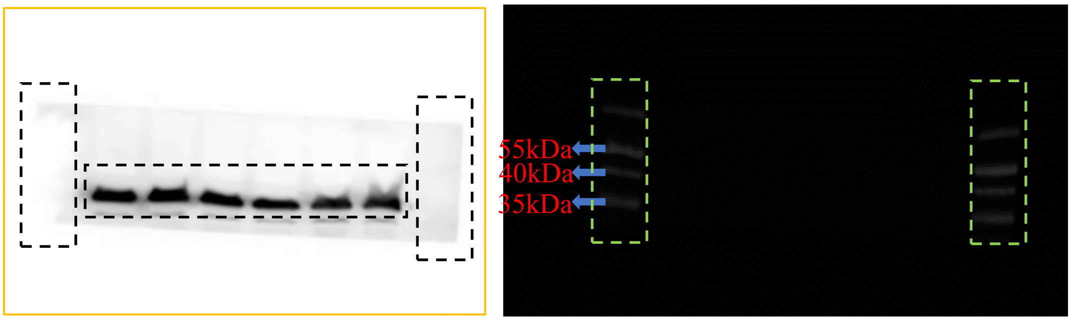

Supplemental Figure S3D related to Figure 6K

MAP4K3 101KDa

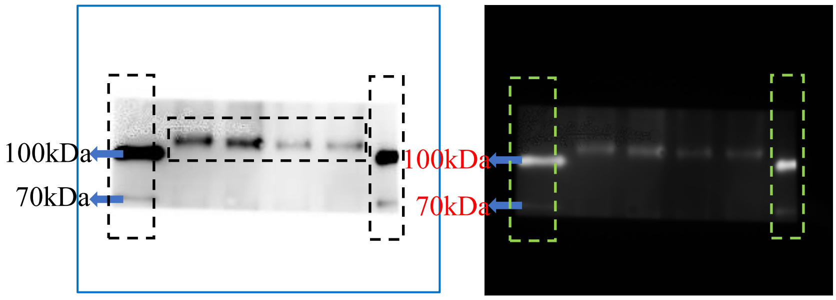

LC3 17KDa, 14KDa

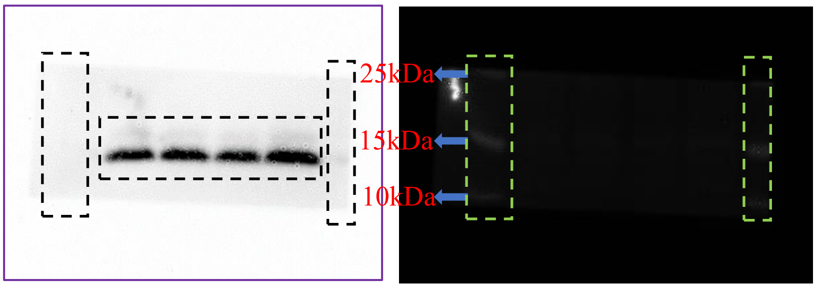

GAPDH 37KDa

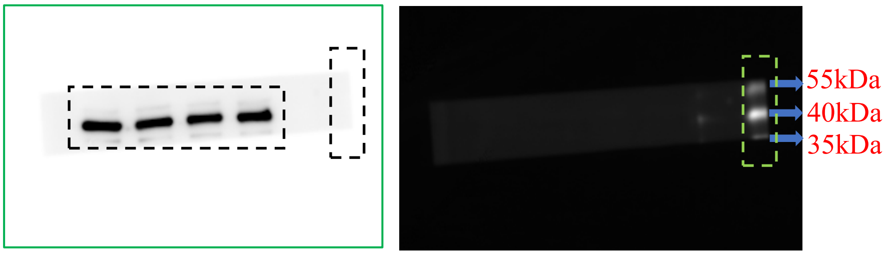

Supplemental Figure S3E related to Figure S1C

CD81 20KDa

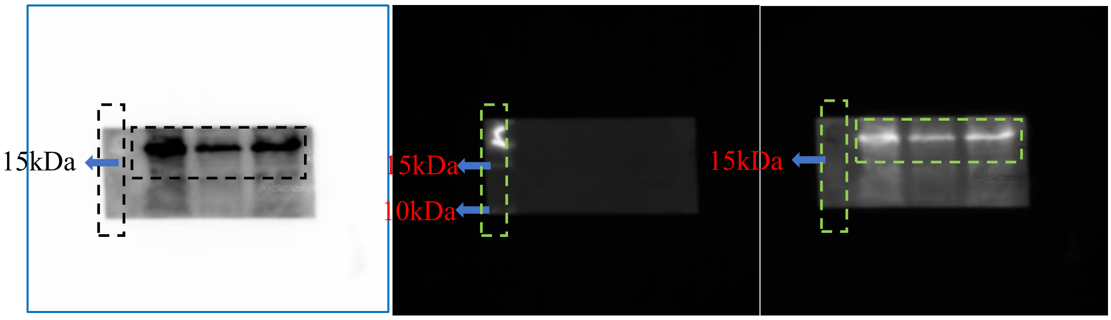

TSG101 50KDa

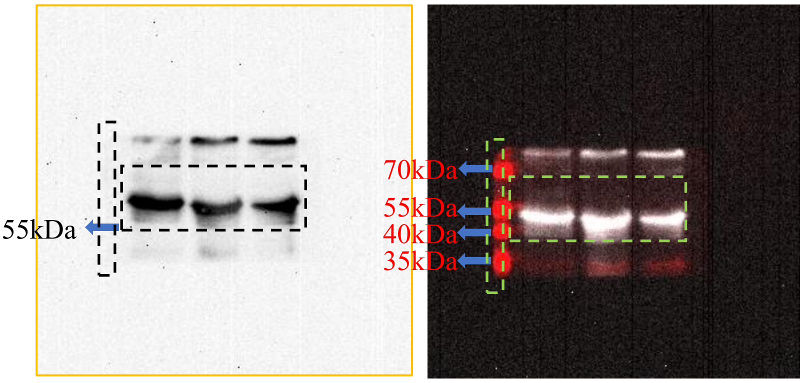

Supplemental Figure S3E related to Figure S2A

STX17 33KDa

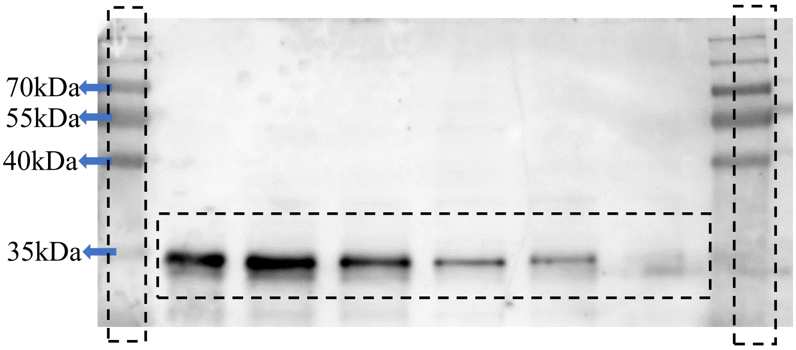

ATG14 65KDa

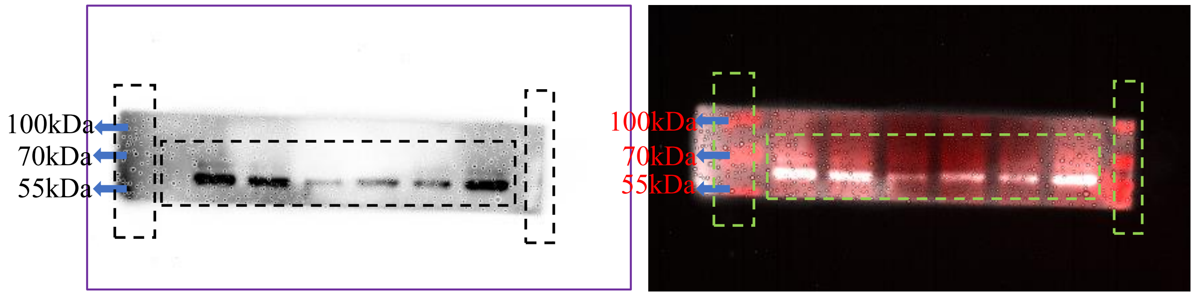

CTSB 27KDa

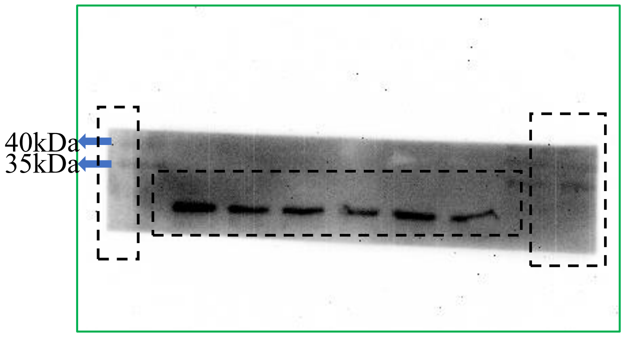

LAMP1 100KDa

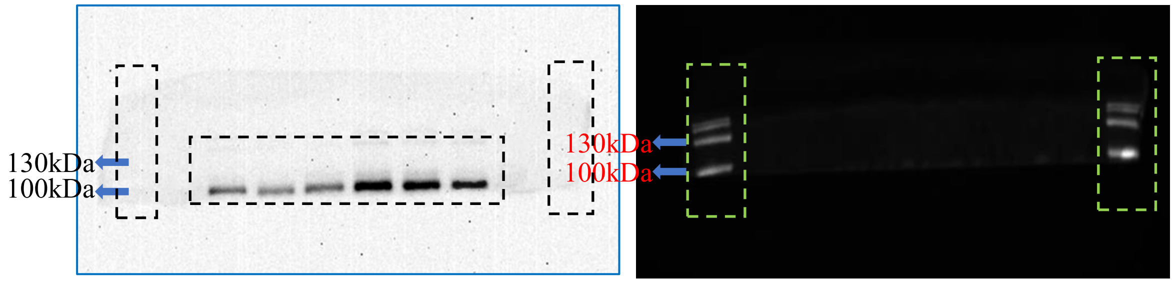

GAPDH-37KDa

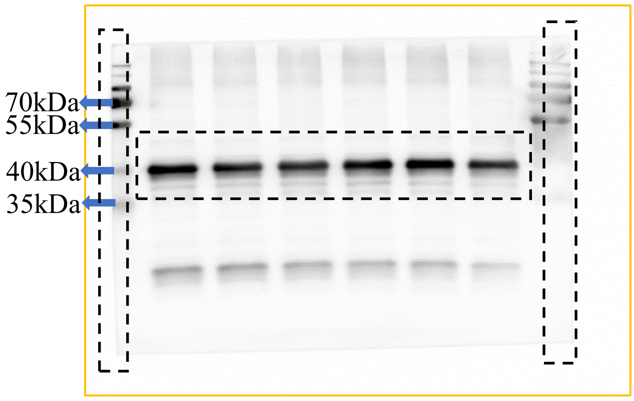

Supplement: Supplementary file 7 — full and uncropped western blots [file 41419_2022_5303_MOESM7_ESM.pdf]
